# A flexible, stretchable system for simultaneous acoustic energy transfer and communication

**DOI:** 10.1126/sciadv.abg2507

**Published:** 2021-09-29

**Authors:** Peng Jin, Ji Fu, Fengle Wang, Yingchao Zhang, Peng Wang, Xin Liu, Yang Jiao, Hangfei Li, Ying Chen, Yinji Ma, Xue Feng

**Affiliations:** 1 AML, Department of Engineering Mechanics, Tsinghua University, Beijing 100084, China.; 2 Center for Flexible Electronics Technology, Tsinghua University, Beijing 100084, China.; 3 Institute of Flexible Electronics Technology of THU Jiaxing, Zhejiang 314000, China.; 4 Qiantang Science and Technology Innovation Center, Hangzhou 310016, China.

## Abstract

The use of implantable medical devices, including cardiac pacemakers and brain pacemakers, is becoming increasingly prevalent. However, surgically replacing batteries owing to their limited lifetime is a drawback of those devices. Such an operation poses a risk to patients—a problem that, to date, has not yet been solved. Furthermore, current devices are large and rigid, potentially causing patient discomfort after implantation. To address this problem, we developed a thin, battery-free, flexible, implantable system based on flexible electronic technology that can not only achieve wireless recharging and communication simultaneously via ultrasound but also perform many current device functions, including in vivo physiological monitoring and cardiac pacing. To prove this, an animal experiment was conducted involving creating a cardiac arrest model and powering the system by ultrasound. The results showed that it automatically detected abnormal heartbeats and responded by electrically stimulating the heart, demonstrating the device’s potential clinical utility for emergent treatment.

## Teaser

A flexible device is capable of simultaneous acoustic energy transfer and communication, for implantable medical application.

## INTRODUCTION

Implantable electronic equipment (IEE) has become vital in the medical field and can perform tasks such as drug delivery and physiological parameter monitoring, as well as function as cardiac and brain pacemakers (*1*–*5*). The need for these devices is well supported by a 2017 report that states that approximately a million pacemakers are implanted each year worldwide (*6*). In addition, implantable glucose sensors can provide accurate, real-time glucose level monitoring without jabbing fingers, which reduces the daily pain that many diabetic individuals must currently endure (*7*).

The power supply for these devices, however, is still one of the greatest challenges, restricting the application of IEE. The batteries used by IEE as its power source are generally reliable and have high power intensity (*8*). Battery usage for IEE, however, has some critical shortcomings, such as the limited lifetime of the battery and the fact that a greater battery capacity requires a larger battery (*9*). One possible solution is the adoption of implantable fuel cell systems that use endogenous substances, such as glucose, to create electricity through an electrochemical reaction. The disadvantage of a low energy–generating rate has greatly restricted its further application for IEE (*10*).

Thus, a wireless power transfer method, with its unique advantages, is distinguished from other proposed energy solutions (*11*–*13*). The energy source of IEE does not require surgical replacement because the wireless power transfer method provides the means to deliver energy through tissue. It could also minimize the size of IEE, as batteries are intrinsically made large to hold sufficient power over time and thus comprise a substantial portion of the IEE bulk (*14*).

Integrating a wireless communication function with IEE has attracted attention recently because it could expand the use of IEE into more clinical applications. For example, for diabetic patients, an implantable device could achieve reactive administration based on continuous glucose monitoring. Real-time, in vivo physiological data monitoring could be realized through collecting data by sensors and sending them to external receivers such as smartphones, thereby allowing a medical diagnosis via the Internet.

Conventional wireless power transfer and communication methods still require optimization for use in IEE. Currently, there are various methods for wireless power transfer (*2*, *13*, *15*), such as inductive power transfer (*16*), radiofrequency (RF) irradiation (*17*), acoustic power transfer (APT) (*18*), optical power transfer (*19*), magnetoelectric power transfer (*20*), and capacitive power transfer (*21*). Here, the first three methods are discussed below (a comparison of our device with other wireless power transfer systems is shown in table S1). Although by far the most widely used method for powering existing medical devices, inductive wireless power transfer provides limited opportunity for taking into account both making small device size and keeping strong energy transfer performance because it relies on strong coupling between the transmitter and receiver coils for efficient energy transfer (*20*). RF approaches, in contrast, can transmit energy through several centimeters of tissue but are limited by tissue absorption and the dimensions of the receiver required to capture sufficient power (*14*, *17*). In contrast, acoustic waves at ultrasound frequencies suffer from substantially lower tissue attenuation and have shorter wavelengths in tissue and may therefore have the potential to operate at greater depths with smaller device dimensions (*17*, *22*). Furthermore, ultrasound is widely accepted for use in medical diagnostics. Hence, APT could be a highly suitable power supply choice for IEE. Traditionally, commercial wireless technologies (e.g., Wi-Fi and Bluetooth) use RF technology for wireless communication. Compared with the acoustic communication method, however, the RF-based wireless communication method is disadvantageous in terms of the potential for miniaturization because RF waves have a larger wavelength than acoustic waves. Acoustic communication techniques are therefore used in a variety of fields, including underwater communications (*23*), airborne ultrasound (*24*), and medical ultrasonic imaging (*25*).

Moreover, currently, most IEEs are made in large sizes and adopt hard structure materials, which can cause discomfort for the patient after implantation. However, by using flexible electronic technologies that comprise emerging design strategies and fabrication techniques to assemble high-performance electronic components in soft, flexible substrates (*26*–*40*), this IEE could be fabricated in slight, flexible, and stretchable forms to reduce the constraint on muscle movement (*41*–*48*), enabled by low-modulus materials and stretchable interconnection structure design (*49*–*51*), with broad medical and industrial applications (*52*–*64*). Recently, state-of-the-art ultrasound-powered devices can substantially reduce the dimensions of implants so that they have decreased implantation risk and show promising implantable medical applications of wireless acoustic energy transfer and communication (*65*–*68*). Sonmezoglu *et al.* developed a miniaturized ultrasound-powered implant (3 mm by 4.5 mm by 1.2 mm) that could wirelessly monitor deep-tissue oxygenation (tested at centimeter-scale depths in sheep) (*69*). Piech *et al.* developed a tiny ultrasound-powered implantable neural stimulator (3.1 mm by 1.9 mm by 0.8 mm) with bidirectional communication function that may facilitate closed-loop neurostimulation for therapeutic interventions (*18*). Many clinical applications, however, have high power requirements that necessitate the use of ultrasonic receivers with larger, centimeter-scale dimensions (*9*). Moreover, state-of-the-art technology cannot yet form flexible devices with high power ability and, in the meanwhile, keeps low constraint on muscle movement, which limits opportunities for implantation and poses a risk of patient discomfort. The device made here uses fractal serpentine copper as interconnection and is encapsulated in a soft polydimethylsiloxane (PDMS) layer, which can be stretchable and match the deformation of biological tissue better.

We therefore developed an implantable acoustic energy transfer and communication device (AECD). Fabricated by using flexible electronic technology, the AECD is soft and comfortable, adapting well to curved human bodies. As shown in fig. S14, the AECD’s stretchability could be up to 20%, which is higher than the typical tensile strain of human skin (<20%) (*70*). We used APT to implement wireless energy transfer and communication, taking into consideration the high biocompatibility of the AECD with the human body. The inner components are fully sealed by PDMS, which has high biocompatibility (*71*), and some scholars have demonstrated that these electronic devices encapsulated by PDMS have high hemocompatibility, low inflammation, and long-term usage potential (*71*–*73*). In addition, the PDMS materials also have strong biodurability, because scholars have tested that implants made of these materials would not fail within 32 years (*74*). Implantable devices using PDMS as encapsulation have also been found in other studies (*16*, *75*). The AECD receiver could also be designed in a small form because of the APT’s short wavelength. Furthermore, to improve the acoustic intensity of energy transfer, we developed a convenient, effective, easy-to-operate ultrasonic focusing method that not only easily accomplishes rapid ultrasonic focusing but also controls the ultrasonic focusing position. This is in contrast to many current acoustic focusing technologies, such as field-programmable gate array technology, which is very efficient but sophisticated and demands high and severe requirements for the hardware environment. As a multifunctional implantable electronic device, the AECD is highly versatile and can work either as a single supplementary power provider for other IEEs or as an independent piece of IEEs with distinctive applications. For example, integrated with specific sensors (e.g., thermal, voltage, pressure, displacement, glucose, and blood oxygen sensors), it can monitor physiology in vivo, providing accurate monitoring of the physical condition in real time. Moreover, after proper programming, the AECD could provide certain special urgent treatments, functioning as a heart pacemaker and nerve stimulator. We have developed a stretchable ultrasonic device capable of ultrasonic energy transfer and communication with significance, such as bringing better comfortableness after the device is implanted, enabling the device to robustly maintain good ultrasonic function under deformation configuration and intelligently monitor the heart, including during emergent treatment.

## RESULTS

### Device design

**Fig. 1.**
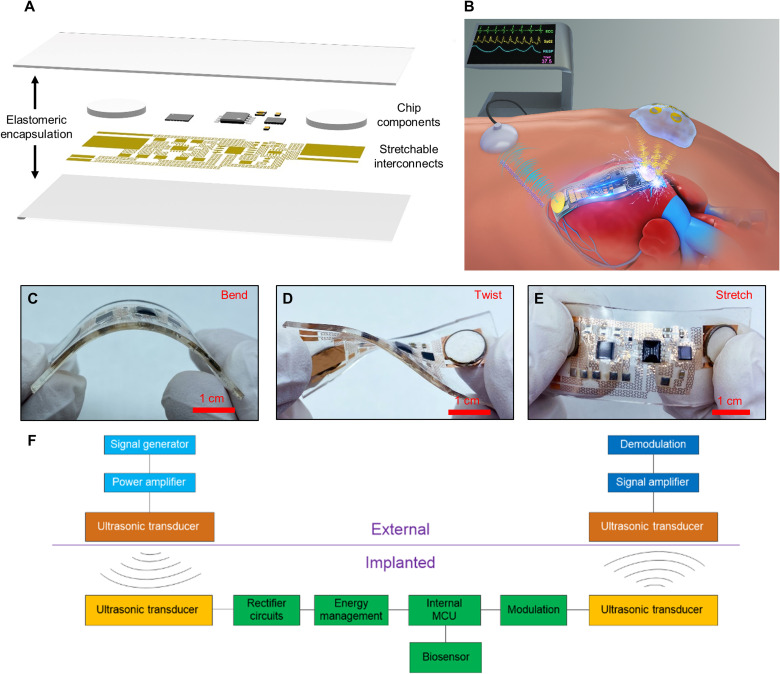
Schematic illustration of the AECD. (**A**) Exploded schematics of the device structure. (**B**) Illustration showing the AECD function—wireless charging and communication based on ultrasound. (**C**) Image of the device in the bent configuration. (**D**) Image of the device in the twisted configuration. (**E**) Image of the device in the stretched configuration. (**F**) System schema for the AECD in use. Photo credit: Peng Jin, Tsinghua University.

As shown in Fig. 1A, the AECD incorporates a fractal serpentine copper pattern placed on a polyimide (PI) pattern layer as the circuit interconnects between chip components. All these components and interconnects are encapsulated in a soft PDMS layer, which makes the AECD soft, flexible, and capable of accommodating most body deformation, such as bending, twisting, and stretching, without altering the operation (Fig. 1, C to E). Furthermore, the flexibility of the AECD was verified by repeated mechanical testing (applying a load 500 times, a bending angle of 80°, and a load frequency of 1 cycle/s; fig. S15). These attributes enable the device to minimize the damage and inflammatory response after implantation into the human body. Although the functional components used here are all rigid, the whole system is still stretchable and flexible (detailed illustration in note S7).

The AECD can simultaneously achieve wireless energy transfer and wireless communication based on ultrasound, as schematically shown in Fig. 1F. To acquire this function, the AECD integrates five modules: an ultrasonic transducer module, a rectifier module, an energy management module, a microprogrammed control unit (MCU), and a sensor module. First, in vivo, the AECD ultrasonic transducer accepts the ultrasound transmitted by an external ultrasonic transducer to produce electrical current, which, in turn, is processed by the rectifier module and the energy management module to power the MCU. After being activated, the MCU collects physiological information through biosensors and controls an ultrasonic transducer to transmit information-coded ultrasound. An external ultrasound capture system can capture this information-coded ultrasound and demodulate it to recover physiological information. Specifically, Fig. 1B demonstrates that the AECD works as an implantable heart monitoring device that wirelessly monitors the heart’s health state and is powered by ultrasound. The system layout and component information are shown in fig. S4. The in-depth ultrasonic energy transfer performance was also explored and illustrated in fig. S5. The system presents admirable energy transfer efficiency (from excitation peak–peak voltage of 18,000 mV to response peak–peak voltage of 4256 mV, with a 5-mm transmission distance, using fat as transmission media) and low energy attenuation (response peak–peak voltage from 4256 to 1876 mV versus transmission distance from 5 to 50 mm under similar excitation conditions). Under different deformation states, the AECD still has strong electrical robustness, which can receive ultrasound and then steadily produce a 3.3-V DC voltage as a power supply (fig. S6). Furthermore, the charging rate of the AECD is explored in fig. S10, according to which the charging time is approximately 5.5 ms to fully charge the energy storage capacitance of the AECD. In addition, the measured power transfer efficiency of this acoustic energy transfer method could be up to 23% at a 5-mm distance, as shown in fig. S13 (pork tissue as transmission media, consumed power at 328 mW, and received power at 75 mW; detailed power calculation is shown in note S4). The received power intensity is 95.5 mW/cm^2^ (the received power is 75 mW, and the area of the receiver ultrasound transducer is 0.785 cm^2^), which is under the safe limit of 720 mW/cm^2^, given by the U.S. Food and Drug Administration (*22*).

### Fabrication process

**Fig. 2.**
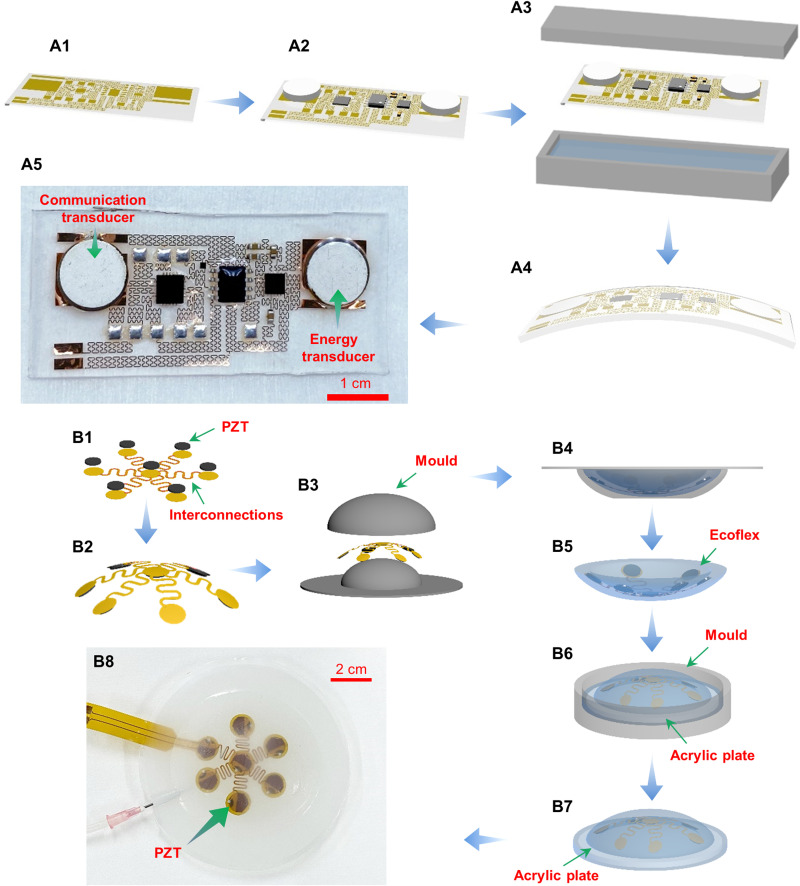
Manufacturing process. (**A**) Steps in manufacturing the AECD. (**B**) Steps in manufacturing the external energy–transmitting equipment. Photo credit: Peng Jin, Tsinghua University.

The manufacturing process for the AECD requires two main steps: first establishing the circuit and then packaging it (Fig. 2). Specially designed external energy-transmitting equipment (EETE) that transmits ultrasound, thereby providing energy to the AECD, was also fabricated.

Fabricating the AECD includes three steps: (i) patterning circuit interconnects on a flexible film and transferring them onto a soft substrate (film circuit) (Fig. 2A1), (ii) bonding chip components on the film circuit (Fig. 2A2), and (iii) encapsulating the film circuit into a soft polylayer (Fig. 2, A3 and A4). The first step starts with coating a polymethylmethacrylate (PMMA) film on a silicon wafer as a sacrificial layer and an ultrathin PI film on the PMMA film by spinning. Then, a metal layer of 500-nm Cu was deposited on the cured PI film by electron beam evaporation. After that, the traditional lithography method is used to etch the metal circuit interconnect pattern. Next, this Cu-PI-PMMA layer was peeled from the silicon wafer and applied to soft cured PDMS. In the second step, screen printing technology was used to fill solder pastes on the metal pattern, and chip components and PZT (lead-zirconate-titanate) transducers were placed on the circuit and bonded by heating to 190°C for approximately 15 s. Furthermore, reactive ion etching is also used to etch the PI film into the same metal pattern to further reduce the bending and tension stiffness of the film circuit. In the third step, the film circuit bonded with chips is then placed inside a mold filled with fluid PDMS and a properly configured catalyzer. The mold was then heated to 70°C and maintained at that temperature for approximately half an hour. After that, the AECD was fabricated and is shown in Fig. 2A5. The PZT soldering connection is shown in fig. S2, and the cured temperature for the PZT is 190°C. After the PZT is cured, there is a slight shift in the resonant frequency from 2 to 1.94 MHz (fig. S3). As shown in fig. S12, the PZT plate fabricated by the high-temperature reflow process (using solder paste, curing at 190°C for 15 s) still has almost the same acoustic performance as the PZT plate fabricated by the low-temperature process (using silver paint, curing at 60°C for 30 min) [almost the same excitation voltage: peak-to-peak (P-P) voltages of 8.94 and 8.95 V, close response voltages: P-P voltages of 3.27 and 3.12 V, respectively].

The procedure for fabricating EETE comprises creating an array of ultrasonic transducers and implanting the completed device into a flexible base. To create the ultrasonic array, an ultrathin, flexible, printed circuit is produced using identical flexible printed circuit technology (Fig. 2B1). Then, reflow soldering technology is used to solder ultrasonic transducers onto the premade circuit in the form of a quincunx (Fig. 2B2). Next, the prefabricated circuit is placed inside the first mold (Fig. 2, B3 and B4) filled with silicone (Ecoflex, Smooth-On Inc., Macungie, PA, USA) as encapsulation, which is cured at 70°C for approximately half an hour. The first curved part of EETE is now ready (Fig. 2B5). An acrylic plate is placed at the bottom of another mold, and then liquid silicone is poured. After that, the curved part of EETE is placed on the mold. Then, the mold was cured at 70°C for half an hour (Fig. 2B7). The completely fabricated EETE is shown in Fig. 2B8. Here, acoustic impedance matching is also considered, and the materials used in EETE have very close acoustic impedance (Ecoflex, 1.91 Mrayl; water, 1.48 Mrayl; Perspex, 3.22 Mrayl), as shown in fig. S9.

### Ultrasonic wireless energy transfer

The AECD achieves wireless power transfer based on an acoustic method: the conversion of mechanical to electrical energy. This energy conversion is performed as follows. First, in vitro, the equipment transmits ultrasound to the AECD receiver. Then, in vivo, the AECD ultrasonic transducer, composed of piezoelectric material, accepts ultrasound and converts it to electrical energy. Because the ultrasonic transducer produces only AC, however, the current must pass through the AECD rectifier module to be transformed to DC. The AECD energy management is responsible for supplying energy to the entire internal electronic system.

**Fig. 3.**
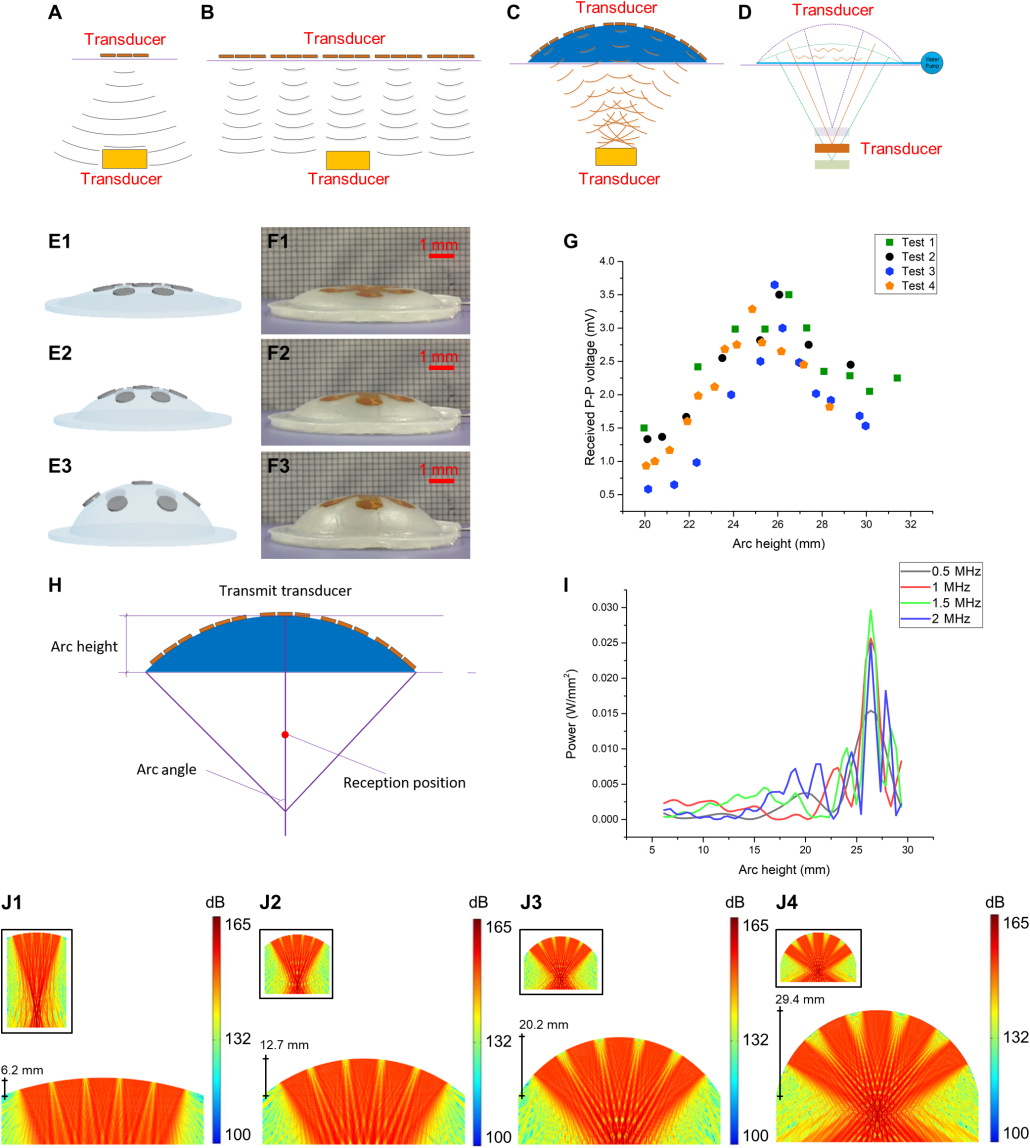
Ultrasonic focusing method achieved by changing the geometry of the flexible base. (**A**) Ultrasonic transmission using a few ultrasonic transducers. (**B**) Ultrasonic transmission by a tiled array of ultrasonic transducers. (**C**) Ultrasonic transmission by an array of ultrasonic transducers placed inside the top part of a flexible base. (**D**) Adjusting the ultrasonic focusing position by changing the curvature of the flexible base upper surface. (**E**) Deformation process of the flexible base while injecting water. (**F**) Deformation process of the flexible base during the experiment. (**G**) Experimental results for the received P-P voltage with different arc heights, normalized by 60 mV, which is the received P-P voltage when the arc height was 0 mm. (**H**) Description of geometric relation variables and reception position. (**I**) Acoustic power in a fixed reception position with different arc heights and ultrasonic transmission frequencies during a simulation. (**J**) Acoustic pressure level distribution during the deformation process of the flexible ultrasonic launcher during a simulation. Photo credit: Peng Jin, Tsinghua University.

A convenient effective ultrasound-focusing technology that takes both flexibility and acoustic intensity into consideration has been developed. The design principle and possible results are shown in Fig. 3. If we use only one ultrasonic transducer with a large surface, acoustic scattering triggers big loss of ultrasonic energy. In addition, the user experiences discomfort because of the transducer rigidity (Fig. 3A). A possible solution is to use many small transducers in an array, although it is obvious that the design of the array could severely affect the efficiency of the power transfer. For example, if a tiled layout is used, as seen in most off-center transducers, the ultrasonic intensity at the receiving end is low. The reason for this is that the acoustic energy, starting from the distant portion of the transducer, attenuates as it moves (much of it not in the central axis of the transducer) toward the receiving end, resulting in a low-energy level arriving at the specific receiving position in the central axis of the array. Thus, combining the contributions of all the small, individual transducers results in overall low total energy because of attenuation (Fig. 3B). Hence, we selected a centralized, symmetrical, curved design so that ultrasound would focus on a specific point in the central line (Fig. 3, C and D).

The array is implanted in a deformable flexible base composed of silicone (Ecoflex, Smooth-On Inc., Macungie, PA, USA) with a cavity inside. The curvature of the upper surface of the base can be changed by injecting water into the base (Fig. 3E), with the focusing position changed as well. Thus, using geometric regulation, it is easy to focus the ultrasound and adjust the focusing position.

Next, a special experiment was conducted to confirm that it is feasible to adjust the ultrasonic focus by changing the geometry of the outer ultrasonic transducer. First, we injected water into the flexible base to enlarge the curvature and arc height of the outer transmission portion. We then recorded the received P-P voltage of a fixed reception position at a depth of 10 mm (ultrasound frequency is 2 MHz). During the experiment, the deformation process of the flexible base was captured by a camera, some frames of which are shown in Fig. 3F. Moreover, experiments have also been performed to characterize the EETE. The experimental results (Fig. 3G) show that, given a specific ultrasonic reception position, there is a best arc height of the flexible base for achieving the best acoustic intensity. The geometric relation variables are shown in Fig. 3H. Moreover, the maximum sound pressure of EETE could be up to 0.17 MPa, measured by using a hydrophone, as shown in fig. S7.

To confirm the technical feasibility of the proposed method—that the ultrasonic focus position can be adjusted by changing the geometry of the flexible base—an acoustic simulation test was carried out. In this simulation, the transmission medium is simplified as a uniform medium (the transmission loss caused by structural material variance is very slight, owing to the similar material acoustic impedance; details in note S8). For this test, the acoustic field of the ultrasonic transmission array was simulated during deformation of the flexible base. We used some assumptions to simplify the simulation. First, the piezoelectric plate used for transmission was simplified by using a linear acoustic source, which is a common simplification for piezoelectric plates. Second, it was assumed that the medium of the acoustic field was continuous and consisted only of water. The simulation results are shown in Fig. 3 (I and J). Figure 3I demonstrates the focus effect of the fixed reception position, in which the received acoustic power increased from approximately 0.002 W/mm^2^ (the arc height is approximately 6.2 mm) to 0.025 W/mm^2^ (the arc height is approximately 26 mm). The same conclusion was reached by using different ultrasonic transmission frequencies (0.5, 1, 1.5, and 2 MHz; Fig. 3I), which agreed with the experimental result that the best arc height was approximately 26 mm for reception at a depth of 10 mm. It was also evident that ultrasound focusing was achieved and that the focusing position could be adjusted by controlling the shape of the ultrasonic transmission device (Fig. 3J). The influence of the receiving angle on the ultrasonic energy transmission is explored in note S9. In addition, acoustic transmission performance comparison between the EETE and a single PZT plate is shown in note S10.

### Ultrasonic wireless communication

The AECD can transmit information-coded ultrasound, thereby achieving ultrasound-based wireless communication. Specifically, in a working state, the MCU controls a biosensor to acquire physiological information and then modulates that information into the corresponding pulse signal. Using the specific modulated pulse signal, the MCU controls an input/output port to stimulate the emissive ultrasonic transducer to transmit ultrasound, which carries internal physiologic information. At the same time, an external receiving ultrasonic transducer accepts the ultrasound and converts it to a voltage signal. Simultaneously, this voltage signal, undergoing amplification and filtering, can be demodulated and recovered by a computer to obtain the measured internal physiologic information.

**Fig. 4.**
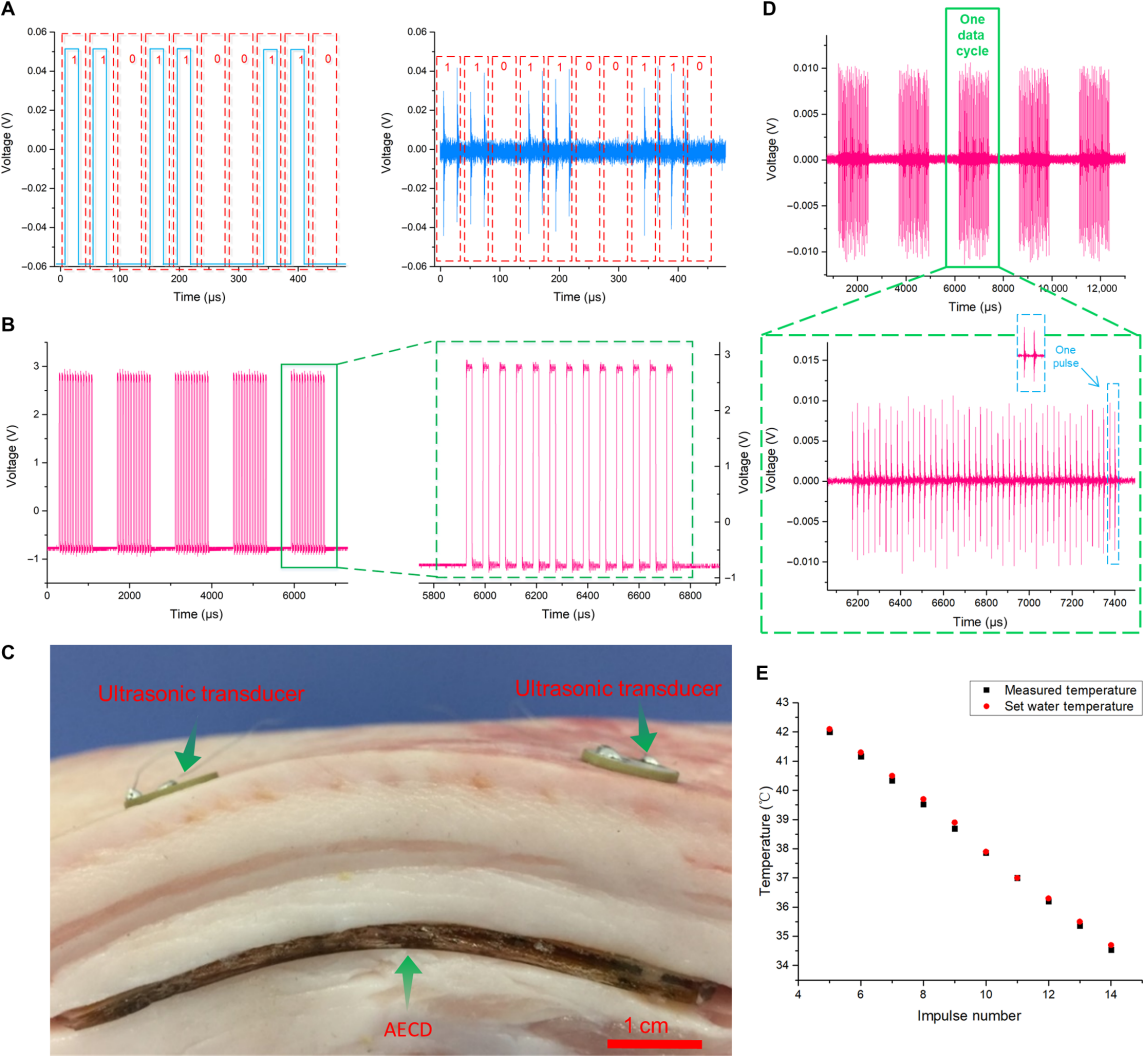
Mechanism and experiment to prove successful ultrasonic wireless communication based on ultrasound. (**A**) Binary modulation method. (**B**) Pulse number modulation method. (**C**) Experiment designed to show that the AECD could accept ultrasound transmitted across biological tissue. (**D**) Temperature information–coded ultrasound showing that the AECD was activated by ultrasound and began to transmit measured temperature data after accepting ultrasound from outside the biological tissue. (**E**) AECD temperature monitoring experiment result. Photo credit: Peng Jin, Tsinghua University.

We use the readings of special signal modulation and demodulation techniques to explain wireless acoustic communication (Fig. 4, A and B). For temperature signal processing, when the MCU acquires data from the temperature sensor, the temperature value is presented in the form of a numerical value, which must be transformed into a pulse signal for the convenience of ultrasonic communication. Here, we present two easy ways to perform modulation and demodulation.

For the first method, with modulation, the numerical temperature value is transformed into binary form. The MCU, according to that binary number, then transmits a particular pulse signal to stimulate the emissive ultrasonic transducer. Specific ultrasound-carried temperature information is then transmitted (Fig. 4A). Next, with demodulation, the received ultrasound is retransformed into a binary number based on the ultrasound waveform. It is then easy to obtain the measured temperature value from the binary number.

For the second method, with modulation, after analog-to-digital (ADC) processing, the MCU receives an integer corresponding to the temperature response from the temperature sensor (the thermoelectrical response of the temperature sensor and the ADC setup is shown in note S1). A mapping relationship is then established between the temperature value in the form of an integer and the pulse number in one data cycle. Specifically, normally, human body temperature is in the range of 35° to 42°C. If in that temperature range, after ADC processing, the MCU would obtain an integer from 205 to 213. To minimize the data cycle size, 200 is subtracted from the obtained integer to be used as the ultimate data. For example, when the temperature is 26.2°C, collected by an ADC port with a resolution of eight bits, the MCU receives an integer of 224. After subtraction, an integer 24 is acquired. The MCU then transmits a pulse signal that contains 24 pulses in one data cycle to stimulate the emissive ultrasonic transducer (Fig. 4B), making the transducer transmit specific ultrasound that carries the temperature data (a more detailed calculation process is shown in note S1). With demodulation, after receiving the specific ultrasound, it is easy to obtain the temperature data based on the established mapping relationship. Each of the two proposed approaches is feasible but has different characteristics. Compared with the second method, the first method provides faster transmission speed with efficient data storage in the binary system. However, these advantages could be eclipsed by being more easily disturbed and having a lower error tolerance. Even a simple packet loss or change in one bit can make the results totally different. Hence, here, we adopt the second method to conduct the experiment.

Here, an experiment was undertaken to directly demonstrate the function of the AECD in simultaneous acoustic energy transfer and communication in a biological environment. In this experiment, as shown in Fig. 4C, we cut the side face of a piece of fresh pork (depth 17 mm) and then buried the AECD inside the pork. The environmental temperature was 26.2°C (measured by a thermocouple thermometer). Here, in this experiment, the required acoustic energy is not large, and the PZT transmission transducer used here is sufficient to power the AECD. Two external ultrasonic transducers were then placed on the upper surface of the piece of pork, facing the corresponding internal ultrasonic transducer (Fig. 4C). Next, one external ultrasonic transducer is stimulated by AC and begins to transmit ultrasound. Subsequently, one internal ultrasonic transducer received the ultrasound and converted it into electrical energy to support the AECD. Then, the AECD started to collect the temperature information and then transmitted the temperature information–coded ultrasound, which was then received by another external ultrasonic transducer (Fig. 4D) (the signal-to-noise ratio of the received ultrasound signal is 16.8192; the calculation is shown in note S3, and the frequency analysis is shown in fig. S8). The maximum data bandwidth here could be at least up to 0.4 kHz (details are given in note S6). Through the second presented demodulation method, the temperature value was easily decoded (26.2°C). Moreover, to explore the AECD temperature monitoring performance in the human body temperature range, a temperature monitoring experiment is conducted. In this experiment, the AECD was put in a constant temperature water bath heating device. The water temperature was set step-by-step from 34.7° to 42.1°C (the step size was approximately 0.7°C). When the water temperature was stable at each set temperature, the set water temperature and the measured temperature were synchronously recorded from the AECD acoustic communication result, as shown in Fig. 4E (the mean relative error of the result was approximately 0.3%, calculated based on Fig. 4E, and the calculation equation is shown in note S2).

### Cardiac pacing experiment

**Fig. 5.**
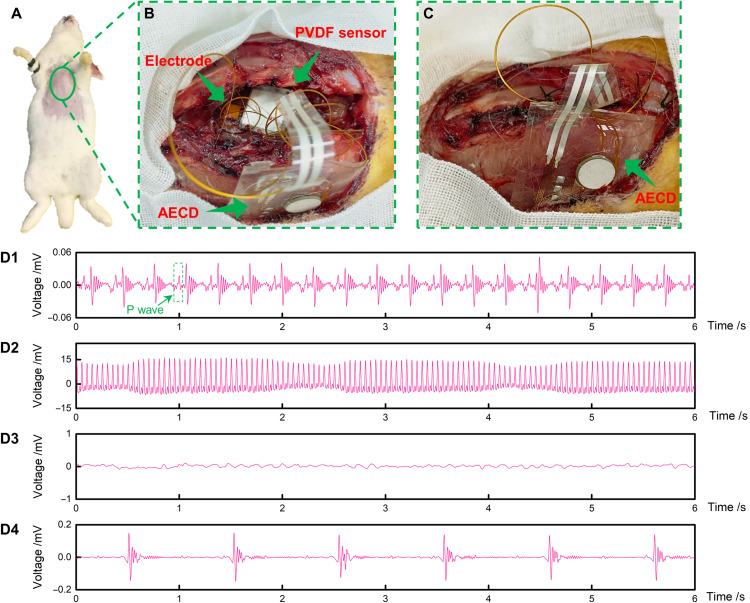
Cardiac pacing experiment to prove the AECD ability to serve as a cardiac pacemaker. (**A**) Experimental animal. (**B**) Fixing the AECD, sensors, and electrodes. The top arrow shows the PVDF, the middle arrow shows the electrode, and the bottom arrow shows the AECD. (**C**) Suturing the rabbit’s chest. (**D1**) Rabbit’s regular heartbeat using ultrasound to power the AECD. (**D2**) Rabbit ECG, when using a high-voltage AC directly to stimulate the heart to cause cardiac arrest. (**D3**) Rabbit ECG to confirm that cardiac arrest was initiated successfully. (**D4**) Rabbit ECG shows that the AECD, powered by ultrasound, successfully detected abnormal heartbeats and stimulated the heart. Photo credit: Peng Jin, Tsinghua University.

The AECD is a versatile type of IEE. With specific design, it can function in many areas, such as acting as a heart pacemaker and nerve stimulator. To prove the AECD ability to function as a heart pacemaker, a cardiac pacing experiment was conducted. The experimental subject was a rabbit (Fig. 5A). The study aimed to prove that the AECD could detect an abnormal heartbeat, here defined as asystole, and then respond by sending a specifically programmed electrical signal to stimulate the heart as an emergent treatment. The heartbeat detection function of the AECD is as follows. It uses a polyvinylidene difluoride (PVDF) sensor, which is an ultrathin piezoelectric film sensor that can collect weak strain signals, thereby monitoring the heartbeat. The AECD MCU periodically and automatically analyzes the heartbeat signal (in this experiment, a normal state was considered at least one heartbeat per second) to determine whether the heartbeat rhythm is normal (signal processing for cardiac arrest detection performed on the AECD). If an abnormal heartbeat occurs, the MCU immediately stimulates the heart via a specific electrical signal. In this experiment, the AECD was implanted inside the subcutaneous tissue of a rabbit’s chest (thus, the ultrasound would not encounter biological tissue with high acoustic impedance, such as pulmonary lobes and bone, in the travel path). The PVDF sensor and stimulating electrode were then fixed on the rabbit’s heart with stitches (Fig. 5B). After fixing these devices, the opened chest was sutured (Fig. 5C). A connection illustration of the stimulating electrode and the PVDF sensor on the AECD is shown in fig. S11A. Detailed device placement in the in vivo experiment is shown in fig. S11B.

The experimental procedure and results are as follows. First, we fixed the PVDF sensor on the rabbit’s heart by sutures and then recorded the electrocardiogram (ECG) and PVDF sensor signals simultaneously using commercial ECG equipment and a data acquisition device. The results showed that the rabbit had a normal heartbeat and ECG (fig. S1A), well matching the strain signal (fig. S1B). In the following, all the ECG signals, including Fig. 5 (D1 to D4), were recorded by commercial ECG equipment (BL-420F, Chendu Techman Soft, Chendu, China), even when electrical stimulation was performed. After setting up all these devices, when the rabbit was in a normal heartbeat, ultrasound was used to power the AECD, and the ECG was recorded through commercial ECG equipment. The results proved that the AECD succeeded in detecting the normal heart rhythm without releasing an electrical stimulus (the ECG signal is shown in Fig. 5D1). Then, after reopening the chest, we used a high-voltage AC to stimulate the heart directly to cause cardiac arrest (ECG signal is shown in Fig. 5D2). The heart went into cardiac arrest after 1 min of stimulation (ECG signal is shown in Fig. 5D3). Again, using ultrasonic transmitting equipment to power the AECD, we obtained an ECG, which showed an electrical pulse signal with a frequency of 1 Hz (ECG signal is shown in Fig. 5D4). In general, the rabbit’s heartbeat frequency is approximately 3 to 4 Hz, and our device is programmed to give electrical simulation at a frequency of 1 Hz when detecting cardiac arrest. Comparing these two signals in Fig. 5 (D1 and D4), the signal in Fig. 5D1 has a frequency of approximately 3 to 4 Hz, matching the normal rabbit heart frequency, but the signal in Fig. 5D4 has a frequency of 1 Hz, matching the AECD electrical stimulation frequency. Moreover, the signal in Fig. 5D1 has a standard p wave (a standard ECG characteristic), while the signal in Fig. 5D4 does not, which means that the signal in Fig. 5D1 is an ECG signal created for a rabbit’s normal heartbeat, while the signal in Fig. 5D4 is not. According to the above analysis, we conclude that the AECD powered by ultrasound has the capacity to detect abnormal heartbeats and then respond with electrical stimulation.

## DISCUSSION

We have developed a universal flexible and stretchable implantable platform based on an acoustic method that could achieve wireless power transfer and wireless communication. The acoustic intensity for wireless power transfer was evidently improved by using a specially designed ultrasonic transmission system (note S10). In addition, the ultrasonic focus position could be controllable by adjusting the geometry of the ultrasonic transmission system. Experiments and acoustic simulations carried out to certify the feasibility of ultrasonic focus produced highly consistent results. To achieve acoustic wireless communication, we devised two types of special signal modulation and demodulation techniques that encoded information by embedding it in ultrasound and later decoding it from the same received ultrasound using an algorithm. However, the AECD flexibility can still be improved further by doing the following: (i) It is better to use an ultrasound transducer array instead of a rigid plate transducer in the AECD, and (ii) adopting smaller and thinner chip components could further help to reduce the device rigidity. Through in vivo animal experiments, we showed that when used as a flexible cardiac pacemaker, the AECD is capable of monitoring the heartbeat state, and when abnormal heartbeat occurs, it could respond by electrically stimulating the heart as emergent treatment. In addition, the flexible form could bring minimal discomfort to the patient, which is especially beneficial for long-term care. Furthermore, compared with traditional battery-based devices, the acoustic wireless power application here could eliminate the risk of secondary surgery for replacing the battery. As a universal IEE platform, the AECD also has various other applications. For example, it could also potentially be used to treat urinary incontinence; if integrated with a strain sensor, the AECD could monitor the bladder function, and when pathological behavior occurs, it could electrically stimulate the bladder sensory afferents to normalize bladder function (*71*). After specific programming, the AECD can undertake any programmed action when facing various physiologic abnormalities.

## MATERIALS AND METHODS

### Fabrication of the flexible acoustic device

An ultralow-energy harvester chip 4.00 mm by 4.00 mm by 0.8 mm (ADP5091ACPZ-1-R7, Analog Devices, Malpitas, CA, USA); a low-loss, full-wave bridge rectifier chip 4.90 mm by 3.0 mm (LTC3588-2, Analog Devices/Linear Technology, Malpitas, CA, USA); an 8-bit AVR microcontroller chip 4.00 mm by 4.00 mm (ATtiny85 V, Atmel, San Jose, CA, USA); an analog temperature sensor chip 0.91 mm by 0.91 mm (LMT70, Texas Instruments, Dallas, TX, USA); and two ultrasound transducers, PZT-5 and PZT-8, were used. A chip mounter (Fineplacer_145_Pico, FineTech, Berlin, Germany) was used to align and place chips on the circuit. The chip and PZT transducers are bonded by reflow soldering technology. Low–melting point solder paste (Sn42Bi58, KELLYSHUN, Shenzhen, China) was used for the bonding chips and PZT transducers on the circuit.

### Fabrication of the ultrasonic focusing launcher

The ultrasonic focusing launcher was manufactured using a casting method. The cast was created by a high-definition three-dimensional printer (ProJet MJP 5600, 3D Systems, Rock Hill, SC, USA) with photosensitive resin material (9400 photosensitive resin). The ultrasonic focusing launcher was made from an ultrasoft, biocompatible silicone layer (Ecoflex 00-30, Smooth-On Inc., Macungie, PA, USA) with a thickness of 1.6 mm. Curing was performed at room temperature for 4 hours. The implanted ultrasound transducers were PZT-5 and PZT-8.

### Acoustic field simulation

The Multiphysics finite element analysis simulation software (Comsol 5.3.1.180) was used to analyze the sonic pressure field of the ultrasonic focusing launcher. For the simulation, the materials used were water and air. Water filled an arc area and a square area right under the arc with a depth of 35 mm. The arc area had an altered central angle with a range of 20° to 90° in 1° steps. The fluid physical model used linear elasticity. The temperature was set at 293.15 K. The ultrasonic transducers were simplified as a linear acoustic source.

### Animal experiments

All animal experiments were performed at Beijing Medical Services Biotechnology (Beijing, China) and approved by the Ethics Committee of Beijing Medical Services Biotechnology (MDSW-2018-018C). New Zealand rabbits (3.0 to 3.3 kg) were anesthetized by intraperitoneal injection of xylazine hydrochloride (5 mg/kg), and anesthesia was maintained with isoflurane (5%)/oxygen. Placed in the supine position, the rabbit was shaved on the chest near the heart. Thoracotomy was then undertaken, exposing the heart. The rabbit subcutaneous tissues were incised by bistoury on the chest, and the AECD was implanted within subcutaneous tissues. The electric stimulation electrode and PVDF sensor were sutured onto the rabbit’s heart. The stimulation electrode was a commercial copper pad made using flexible printed circuit (FPC) technology. PVDF is a commercial piezoelectric thin film sensor (American Measurement Specialties, Fairfield, NJ, USA). A biological data acquisition and stimulation system (BL-420F, Chendu Techman Soft, Chendu, China) was used to record the ECG. Before the AECD was implanted, a single PVDF was sutured onto the heart. A data acquisition card (National Instruments Corp., Austin, TX, USA) was used to record the PVDF signal to prove the use of PVDF as a heartbeat rhythm monitor.

## Supplementary Materials

### This PDF file includes:

Supplementary Notes S1 to S10 Figs. S1 to S19 Table S1 References

## References

[1] E. H. Ledet, D. DʼLima, P. Westerhoff, J. A. Szivek, R. A. Wachs, G. Bergmann, Implantable sensor technology: From research to clinical practice. J. Am. Acad. Orthop. Surg. 20, 383–392 (2012). 22661568 10.5435/JAAOS-20-06-383

[2] J. Lueke, W. A. Moussa, MEMS-based power generation techniques for implantable biosensing applications. Sensors 11, 1433–1460 (2011). 22319362 10.3390/s110201433 PMC3274013

[3] P. Gerrish, E. Herrmann, L. Tyler, K. Walsh, Challenges and constraints in designing implantable medical ICs. IEEE Trans. Device Mater. Reliab. 5, 435–444 (2005).

[4] P. R. Troyk, G. A. DeMichele, D. A. Kerns, R. F. Weir, in *2007 Annual International Conference of the IEEE Engineering in Medicine and Biology Society, Vols 1–16 Proceedings of Annual International Conference of the IEEE Engineering in Medicine and Biology Society* (IEEE, 2007), pp. 1730–1733. 10.1109/IEMBS.2007.4352644 18002310

[5] K. D. Wise, D. J. Anderson, J. F. Hetke, D. R. Kipke, K. Najafi, Wireless implantable microsystems: High-density electronic interfaces to the nervous system. Proc. IEEE 92, 76–97 (2004).

[6] F. V. Y. Tjong, V. Y. Reddy, Permanent leadless cardiac pacemaker therapy: A comprehensive review. Circulation 135, 1458–1470 (2017). 28396380 10.1161/CIRCULATIONAHA.116.025037

[7] A. Guiseppi-Elie, S. Brahim, G. Slaughter, K. R. Ward, Design of a subcutaneous implantable biochip for monitoring of glucose and lactate. IEEE Sens. J. 5, 345–355 (2005).

[8] D. C. Bock, A. C. Marschilok, K. J. Takeuchi, E. S. Takeuchi, Batteries used to power implantable biomedical devices. Electrochim. Acta 84, 155–164 (2012). 10.1016/j.electacta.2012.03.057 PMC3811938 24179249

[9] F. Akhtar, M. H. Rehmani, Energy replenishment using renewable and traditional energy resources for sustainable wireless sensor networks: A review. Renew. Sustain. Energy Rev. 45, 769–784 (2015).

[10] S. Kerzenmacher, J. Ducree, R. Zengerle, F. von Stetten, Energy harvesting by implantable abiotically catalyzed glucose fuel cells. J. Power Sources 182, 1–17 (2008).

[11] M. A. Hannan, S. Mutashar, S. A. Samad, A. Hussain, Energy harvesting for the implantable biomedical devices: Issues and challenges. Biomed. Eng. Online 13, 79 (2014). 24950601 10.1186/1475-925X-13-79 PMC4075616

[12] A. Ben Amar, A. B. Kouki, H. Cao, Power approaches for implantable medical devices. Sensors 15, 28889–28914 (2015). 26580626 10.3390/s151128889 PMC4701313

[13] A. Kim, M. Ochoa, R. Rahimi, B. Ziaie, New and emerging energy sources for implantable wireless microdevices. IEEE Access 3, 89–98 (2015).

[14] S. O’Driscoll, A. S. Y. Poon, T. H. Meng, Wireless power transmission for implantable medical devices. U.S. Patent 08634928 (2014). 10.1109/IEMBS.2007.4353634 18003300

[15] T. Stuart, L. Cai, A. Burton, P. Gutruf, Wireless and battery-free platforms for collection of biosignals. Biosens. Bioelectron. 178, 113007 (2021). 33556807 10.1016/j.bios.2021.113007 PMC8112193

[16] A. D. Mickle, S. M. Won, K. N. Noh, J. Yoon, K. W. Meacham, Y. Xue, L. A. McIlvried, B. A. Copits, V. K. Samineni, K. E. Crawford, D. H. Kim, P. Srivastava, B. H. Kim, S. Min, Y. Shiuan, Y. Yun, M. A. Payne, J. Zhang, H. Jang, Y. Li, H. H. Lai, Y. Huang, S.-I. Park, R. W. Gereau IV, J. A. Rogers, A wireless closed-loop system for optogenetic peripheral neuromodulation. Nature 565, 361–365 (2019). 30602791 10.1038/s41586-018-0823-6 PMC6336505

[17] J. S. Ho, A. J. Yeh, E. Neofytou, S. Kim, Y. Tanabe, B. Patlolla, R. E. Beygui, A. S. Y. Poon, Wireless power transfer to deep-tissue microimplants. Proc. Natl. Acad. Sci. U.S.A. 111, 7974–7979 (2014). 24843161 10.1073/pnas.1403002111 PMC4050616

[18] D. K. Piech, B. C. Johnson, K. Shen, M. M. Ghanbari, K. Y. Li, R. M. Neely, J. E. Kay, J. M. Carmena, M. M. Maharbiz, R. Muller, A wireless millimetre-scale implantable neural stimulator with ultrasonically powered bidirectional communication. Nat. Biomed. Eng. 4, 207–222 (2020). 32076132 10.1038/s41551-020-0518-9

[19] S. Lee, A. J. Cortese, A. P. Gandhi, E. R. Agger, P. L. McEuen, A. C. Molnar, A 250μm x 57μm microscale opto-electronically transduced electrodes (MOTEs) for neural recording. IEEE Trans. Biomed. Circuits Syst. 12, 1256–1266 (2018b). 30334768 10.1109/TBCAS.2018.2876069 PMC6338085

[20] W. Xiaojuan, L. Jing, Power sources and electrical recharging strategies for implantable medical devices. Front. Energy 2, 1–13 (2008).

[21] Y. Wu, Q. Chen, X. Ren, Z. Zhang, Efficiency optimization based parameter design method for the capacitive power transfer system. IEEE Trans. Power Electron. 36, 8774–8785 (2021).

[22] G. E. Santagati, T. Melodia, Experimental evaluation of impulsive ultrasonic intra-body communications for implantable biomedical devices. IEEE Trans. Mob. Comput. 16, 367–380 (2017).

[23] M. Chitre, S. Shahabudeen, L. Freitag, M. Stojanovic, I. Mts, in *Oceans 2008* (IEEE , 2008), pp. 1–10.

[24] T. Dahl, J. L. Ealo, H. J. Bang, S. Holm, P. Khuri-Yakub, Applications of airborne ultrasound in human-computer interaction. Ultrasonics 54, 1912–1921 (2014). 24974162 10.1016/j.ultras.2014.04.008

[25] F. I. Thurston, H. E. Melton, Biomedical ultrasonics, in *IEEE Transactions on Industrial Electronics and Control Instrumentation* (IEEE, 1970), pp. 167–172.

[26] W. Pang, X. Cheng, H. Zhao, X. Guo, Z. Ji, G. Li, Y. Liang, Z. Xue, H. Song, F. Zhang, Z. Xu, L. Sang, W. Huang, T. Li, Y. H. Zhang, Electro-mechanically controlled assembly of reconfigurable 3D mesostructures and electronic devices based on dielectric elastomer platforms. Natl. Sci. Rev. 7, 342–354 (2020). 34692050 10.1093/nsr/nwz164 PMC8288899

[27] J. Song, X. Feng, Y. Huang, Mechanics and thermal management of stretchable inorganic electronics. Natl. Sci. Rev. 3, 128–143 (2016). 27547485 10.1093/nsr/nwv078 PMC4991896

[28] M. Cai, S. Nie, Y. Du, C. Wang, J. Song, Soft elastomers with programmable stiffness as strain-isolating substrates for stretchable electronics. ACS Appl. Mater. Interfaces 11, 14340–14346 (2019). 30938975 10.1021/acsami.9b01551

[29] J. Zhao, Y. Zhang, X. Li, M. Shi, An improved design of the substrate of stretchable gallium arsenide photovoltaics. J. Appl. Mech. 86, 031009 (2019).

[30] S. Yin, Y. Su, A traction-free model for the tensile stiffness and bending stiffness of laminated ribbons of flexible electronics. J. Appl. Mech. 86, 051011 (2019).

[31] Y. Zhang, N. Zheng, Y. Cao, F. Wang, P. Wang, Y. Ma, B. Lu, G. Hou, Z. Fang, Z. Liang, M. Yue, Y. Li, Y. Chen, J. Fu, J. Wu, T. Xie, X. Feng, Climbing-inspired twining electrodes using shape memory for peripheral nerve stimulation and recording. Sci. Adv. 5, eaaw1066 (2019). 31086809 10.1126/sciadv.aaw1066 PMC6505533

[32] H. Hu, X. Zhu, C. Wang, L. Zhang, X. Li, S. Lee, Z. Huang, R. Chen, Z. Chen, C. Wang, Y. Gu, Y. Chen, Y. Lei, T. Zhang, N. H. Kim, Y. Guo, Y. Teng, W. Zhou, Y. Li, A. Nomoto, S. Sternini, Q. Zhou, M. Pharr, F. L. di Scalea, S. Xu, Stretchable ultrasonic transducer arrays for three-dimensional imaging on complex surfaces. Sci. Adv. 4, eaar3979 (2018). 29740603 10.1126/sciadv.aar3979 PMC5938227

[33] J. Koo, S. B. Kim, Y. S. Choi, Z. Xie, A. J. Bandodkar, J. Khalifeh, Y. Yan, H. Kim, M. K. Pezhouh, K. Doty, G. Lee, Y.-Y. Chen, S. M. Lee, D. D’Andrea, K. Jung, K. H. Lee, K. Li, S. Jo, H. Wang, J.-H. Kim, J. Kim, S.-G. Choi, W. J. Jang, Y. S. Oh, I. Park, S. S. Kwak, J.-H. Park, D. Hong, X. Feng, C.-H. Lee, A. Banks, C. Leal, H. M. Lee, Y. Huang, C. K. Franz, W. Z. Ray, M. M. Ewan, S.-K. Kang, J. A. Rogers, Wirelessly controlled, bioresorbable drug delivery device with active valves that exploit electrochemically triggered crevice corrosion. Sci. Adv. 6, eabb1093 (2020). 32923633 10.1126/sciadv.abb1093 PMC7455185

[34] H. Jeong, J. A. Rogers, S. Xu, Continuous on-body sensing for the COVID-19 pandemic: Gaps and opportunities. Sci. Adv. 6, eabd4794 (2020). 32917604 10.1126/sciadv.abd4794 PMC7467694

[35] D.-H. Kim, R. Ghaffari, N. Lu, J. A. Rogers, Flexible and stretchable electronics for biointegrated devices. Annu. Rev. Biomed. Eng. 14, 113–128 (2012). 22524391 10.1146/annurev-bioeng-071811-150018

[36] S. Choi, H. Lee, R. Ghaffari, T. Hyeon, D.-H. Kim, Recent advances in flexible and stretchable bio-electronic devices integrated with nanomaterials. Adv. Mater. 28, 4203–4218 (2016). 26779680 10.1002/adma.201504150

[37] N. Lu, D.-H. Kim, Flexible and stretchable electronics paving the way for soft robotics. Soft Robot. 1, 53–62 (2014).

[38] C. Choi, J. Leem, M. S. Kim, A. Taqieddin, C. Cho, K. W. Cho, G. J. Lee, H. Seung, H. J. Bae, Y. M. Song, T. Hyeon, N. R. Aluru, S. W. Nam, D.-H. Kim, Curved neuromorphic image sensor array using a MoS_2_-organic heterostructure inspired by the human visual recognition system. Nat. Commun. 11, 5934 (2020). 33230113 10.1038/s41467-020-19806-6 PMC7683533

[39] G. D. Cha, T. Kang, S. Baik, D. Kim, S. H. Choi, T. Hyeon, D.-H. Kim, Advances in drug delivery technology for the treatment of glioblastoma multiforme. J. Control. Release 328, 350–367 (2020). 32896613 10.1016/j.jconrel.2020.09.002

[40] K. W. Cho, W. H. Lee, B.-S. Kim, D.-H. Kim, Sensors in heart-on-a-chip: A review on recent progress. Talanta 219, 121269 (2020). 32887159 10.1016/j.talanta.2020.121269

[41] S. Yao, A. Myers, A. Malhotra, F. Lin, A. Bozkurt, J. F. Muth, Y. Zhu, A wearable hydration sensor with conformal nanowire electrodes. Adv. Healthc. Mater. 6, 1601159 (2017). 10.1002/adhm.201601159 28128888

[42] S. Yao, P. Swetha, Y. Zhu, Nanomaterial-enabled wearable sensors for healthcare. Adv. Healthc. Mater. 7, 1700889 (2018). 10.1002/adhm.201700889 29193793

[43] S. Niu, N. Matsuhisa, L. Beker, J. Li, S. Wang, J. Wang, Y. Jiang, X. Yan, Y. Yun, W. Burnett, A. S. Y. Poon, J. B.-H. Tok, X. Chen, Z. Bao, A wireless body area sensor network based on stretchable passive tags. Nat. Electron. 2, 361–368 (2019).

[44] M. L. Scarpello, D. Kurup, H. Rogier, D. Vande Ginste, F. Axisa, J. Vanfleteren, W. Joseph, L. Martens, G. Vermeeren, Design of an implantable slot dipole conformal flexible antenna for biomedical applications. IEEE Trans. Antennas Propag. 59, 3556–3564 (2011).

[45] Z. Xie, R. Avila, Y. Huang, J. A. Rogers, Flexible and stretchable antennas for biointegrated electronics. Adv. Mater. 32, 1902767 (2019). 10.1002/adma.201902767 31490582

[46] Z. Xie, B. Ji, Q. Huo, Mechanics design of stretchable near field communication antenna with serpentine wires. J. Appl. Mech. 85, 045001 (2018).

[47] K.-G. Lim, S. Ahn, Y.-H. Kim, Y. Qi, T.-W. Lee, Universal energy level tailoring of self-organized hole extraction layers in organic solar cells and organic-inorganic hybrid perovskite solar cells. Energ. Environ. Sci. 9, 932–939 (2016).

[48] H. Kim, K.-G. Lim, T.-W. Lee, Planar heterojunction organometal halide perovskite solar cells: Roles of interfacial layers. Energ. Environ. Sci. 9, 12–30 (2016).

[49] Z. Huang, Y. Hao, Y. Li, H. Hu, C. Wang, A. Nomoto, T. Pan, Y. Gu, Y. Chen, T. Zhang, W. Li, Y. Lei, N. H. Kim, C. Wang, L. Zhang, J. W. Ward, A. Maralani, X. Li, M. F. Durstock, A. Pisano, Y. Lin, S. Xu, Three-dimensional integrated stretchable electronics. Nat. Electron. 1, 473–480 (2018).

[50] J. W. Lee, R. Xu, S. Lee, K. I. Jang, Y. Yang, A. Banks, K. J. Yu, J. Kim, S. Xu, S. Ma, S. W. Jang, P. Won, Y. Li, B. H. Kim, J. Y. Choe, S. Huh, Y. H. Kwon, Y. Huang, U. Paik, J. A. Rogers, Soft, thin skin-mounted power management systems and their use in wireless thermography. Proc. Natl. Acad. Sci. U.S.A. 113, 6131–6136 (2016). 27185907 10.1073/pnas.1605720113 PMC4896718

[51] S. Xu, Y. Zhang, L. Jia, K. E. Mathewson, K. I. Jang, J. Kim, H. Fu, X. Huang, P. Chava, R. Wang, S. Bhole, L. Wang, Y. J. Na, Y. Guan, M. Flavin, Z. Han, Y. Huang, J. A. Rogers, Soft microfluidic assemblies of sensors, circuits, and radios for the Skin. Science 344, 70–74 (2014). 24700852 10.1126/science.1250169

[52] D.-H. Kim, J. Viventi, J. J. Amsden, J. Xiao, L. Vigeland, Y.-S. Kim, J. A. Blanco, B. Panilaitis, E. S. Frechette, D. Contreras, D. L. Kaplan, F. G. Omenetto, Y. Huang, K.-C. Hwang, M. R. Zakin, B. Litt, J. A. Rogers, Dissolvable films of silk fibroin for ultrathin conformal bio-integrated electronics. Nat. Mater. 9, 511–517 (2010). 20400953 10.1038/nmat2745 PMC3034223

[53] Y. F. Yin, M. Li, Y. H. Li, J. Z. Song, Skin pain sensation of epidermal electronic device/skin system considering non-Fourier heat conduction. J. Mech. Phys. Solids 138, 103927 (2020).

[54] J. P. Zhang, Y. H. Li, Y. F. Xing, Theoretical and experimental investigations of transient thermo-mechanical analysis on flexible electronic devices. Int. J. Mech. Sci. 160, 192–199 (2019).

[55] Y. Liu, L. Zhao, R. Avila, C. Yiu, T. Wong, Y. Chan, K. Yao, D. Li, Y. Zhang, W. Li, Z. Xie, X. Yu, Epidermal electronics for respiration monitoring via thermo-sensitive measuring. Mater. Today Phys. 13, 100199 (2020).

[56] S. Xu, Y. Zhang, J. Cho, J. Lee, X. Huang, L. Jia, J. A. Fan, Y. Su, J. Su, H. Zhang, H. Cheng, B. Lu, C. Yu, C. Chuang, T.-i. Kim, T. Song, K. Shigeta, S. Kang, C. Dagdeviren, I. Petrov, P. V. Braun, Y. Huang, U. Paik, J. A. Rogers, Stretchable batteries with self-similar serpentine interconnects and integrated wireless recharging systems. Nat. Commun. 4, 1543 (2013). 23443571 10.1038/ncomms2553

[57] Y. Kim, A. Chortos, W. Xu, Y. Liu, J. Y. Oh, D. Son, J. Kang, A. M. Foudeh, C. Zhu, Y. Lee, S. Niu, J. Liu, R. Pfattner, Z. Bao, T. W. Lee, A bioinspired flexible organic artificial afferent nerve. Science 360, 998–1003 (2018). 29853682 10.1126/science.aao0098

[58] T. Wang, H. Yang, D. Qi, Z. Liu, P. Cai, H. Zhang, X. Chen, Mechano-based transductive sensing for wearable healthcare. Small 14, 1702933 (2018). 10.1002/smll.201702933 29359885

[59] Y. J. Ma, Y. C. Zhang, S. S. Cai, Z. Y. Han, X. Liu, F. L. Wang, Y. Cao, Z. H. Wang, H. F. Li, Y. H. Chen, X. Feng, Flexible hybrid electronics for digital healthcare. Adv. Mater. 32, 1902062 (2020). 10.1002/adma.201902062 31243834

[60] Y. J. Ma, J. Choi, A. Hourlier-Fargette, Y. G. Xue, H. U. Chung, J. Y. Lee, X. F. Wang, Z. Q. Xie, D. Kang, H. L. Wang, S. Han, S. K. Kang, Y. Kang, X. Yu, M. J. Slepian, M. S. Raj, J. B. Model, X. Feng, R. Ghaffari, J. A. Rogers, Y. Huang, Relation between blood pressure and pulse wave velocity for human arteries. Proc. Natl. Acad. Sci. U.S.A. 115, 11144–11149 (2018). 30322935 10.1073/pnas.1814392115 PMC6217416

[61] H. Li, Y. Ma, Z. Liang, Z. Wang, Y. Cao, Y. Xu, H. Zhou, B. Lu, Y. Chen, Z. Han, S. Cai, X. Feng, Wearable skin-like optoelectronic systems with suppression of motion artifact for cuff-less continuous blood pressure monitor. Natl. Sci. Rev. 7, 849–862 (2020). 34692108 10.1093/nsr/nwaa022 PMC8288864

[62] Y. Chen, S. Lu, S. Zhang, Y. Li, Z. Qu, Y. Chen, B. Lu, X. Wang, X. Feng, Skin-like biosensor system via electrochemical channels for noninvasive blood glucose monitoring. Sci. Adv. 3, e1701629 (2017). 29279864 10.1126/sciadv.1701629 PMC5738229

[63] H. Li, Y. Xu, X. Li, Y. Chen, Y. Jiang, C. Zhang, B. Lu, J. Wang, Y. Ma, Y. Chen, Y. Huang, M. Ding, H. Su, G. Song, Y. Luo, X. Feng, Epidermal inorganic optoelectronics for blood oxygen measurement. Adv. Healthc. Mater. 6, 1601013 (2017). 10.1002/adhm.201601013 28244272

[64] C. Wang, X. Li, H. Hu, L. Zhang, Z. Huang, M. Lin, Z. Zhang, Z. Yin, B. Huang, H. Gong, S. Bhaskaran, Y. Gu, M. Makihata, Y. Guo, Y. Lei, Y. Chen, C. Wang, Y. Li, T. Zhang, Z. Chen, A. P. Pisano, L. Zhang, Q. Zhou, S. Xu, Monitoring of the central blood pressure waveform via a conformal ultrasonic device. Nat. Biomed. Eng. 2, 687–695 (2018). 30906648 10.1038/s41551-018-0287-x PMC6428206

[65] M. J. Weber, Y. Yoshihara, A. Sawaby, J. Charthad, T. C. Chang, A. Arbabian, A miniaturized single-transducer implantable pressure sensor with time-multiplexed ultrasonic data and power links. IEEE J. Solid-State Circuits 53, 1089–1101 (2018).

[66] M. M. Ghanbari, D. K. Piech, K. Shen, S. F. Alamouti, C. Yalcin, B. C. Johnson, J. M. Carmena, M. M. Maharbiz, R. Muller, A sub-mm 3 ultrasonic free-floating implant for multi-mote neural recording. IEEE J. Solid-State Circuits 54, 3017–3030 (2019).

[67] C. Shi, T. Costa, J. Elloian, Y. Zhang, K. L. Shepard, A 0.065-mm^3^ monolithically-integrated ultrasonic wireless sensing mote for real-time physiological temperature monitoring. IEEE Trans. Biomed. Circuits Syst. 14, 412–424 (2020). 32012022 10.1109/TBCAS.2020.2971066

[68] R. Hinchet, H. J. Yoon, H. Ryu, M. K. Kim, E. K. Choi, D. S. Kim, S. W. Kim, Transcutaneous ultrasound energy harvesting using capacitive triboelectric technology. Science 365, 491–494 (2019). 31371614 10.1126/science.aan3997

[69] S. Sonmezoglu, J. R. Fineman, E. Maltepe, M. M. Maharbiz, Monitoring deep-tissue oxygenation with a millimeter-scale ultrasonic implant. Nat. Biotechnol. 39, 855–864 (2021). 33782610 10.1038/s41587-021-00866-y

[70] V. Arumugam, M. Naresh, R. Sanjeevi, Effect of strain rate on the fracture behaviour of skin. J. Biosci. 19, 307–313 (1994).

[71] Z. Jia, C. Yang, J. Jiao, X. Li, D. Zhu, Y. Yang, J. Yang, Y. Che, Y. Lu, X. Feng, Rhein and polydimethylsiloxane functionalized carbon/carbon composites as prosthetic implants for bone repair applications. Biomed. Mater. 12, 045004 (2017). 28425918 10.1088/1748-605X/aa6e27

[72] M. C. Bélanger, Y. Marois, Hemocompatibility, biocompatibility, inflammatory and in vivo studies of primary reference materials low-density polyethylene and polydimethylsiloxane: A review. J. Biomed. Mater. Res. 58, 467–477 (2001). 11505420 10.1002/jbm.1043

[73] W. Qian, X. Hu, W. He, R. Zhan, M. Liu, D. Zhou, Y. Huang, X. Hu, Z. Wang, G. Fei, J. Wu, M. Xing, H. Xia, G. Luo, Polydimethylsiloxane incorporated with reduced graphene oxide (rGO) sheets for wound dressing application: Preparation and characterization. Colloids Surf. B Biointerfaces 166, 61–71 (2018). 29544129 10.1016/j.colsurfb.2018.03.008

[74] H. J. Brandon, K. L. Jerina, C. J. Wolf, V. L. Young, Biodurability of retrieved silicone gel breast implants. Plast. Reconstr. Surg. 111, 2295–2306 (2003). 12794472 10.1097/01.PRS.0000060795.16982.1C

[75] P. Gutruf, R. T. Yin, K. B. Lee, J. Ausra, J. A. Brennan, Y. Qiao, Z. Xie, R. Peralta, O. Talarico, A. Murillo, S. W. Chen, J. P. Leshock, C. R. Haney, E. A. Waters, C. Zhang, H. Luan, Y. Huang, G. Trachiotis, I. R. Efimov, J. A. Rogers, Wireless, battery-free, fully implantable multimodal and multisite pacemakers for applications in small animal models. Nat. Commun. 10, 5742 (2019). 31848334 10.1038/s41467-019-13637-w PMC6917818

[76] V. T. Rathod, A review of acoustic impedance matching techniques for piezoelectric sensors and transducers. Sensors 20, 4051 (2020). 32708159 10.3390/s20144051 PMC7411934

[77] B. H. Waters, A. P. Sample, P. Bonde, J. R. Smith, Powering a ventricular assist device (VAD) with the free-range resonant electrical energy delivery (FREE-D) system. Proc. IEEE 100, 138–149 (2012).

[78] A. Abid, J. M. O’Brien, T. Bensel, C. Cleveland, L. Booth, B. R. Smith, R. Langer, G. Traverso, Wireless power transfer to millimeter-sized gastrointestinal electronics validated in a swine model. Sci. Rep. 7, 46745 (2017). 28447624 10.1038/srep46745 PMC5406829

[79] Y. Shih, T. Shen, B. Otis, A 2.3 μ W wireless intraocular pressure/temperature monitor. IEEE J. Solid-State Circuits 46, 2592–2601 (2011).

[80] H. U. Chung, B. H. Kim, J. Y. Lee, J. Lee, Z. Xie, E. M. Ibler, K. H. Lee, A. Banks, J. Y. Jeong, J. Kim, C. Ogle, D. Grande, Y. Yu, H. Jang, P. Assem, D. Ryu, J. W. Kwak, M. Namkoong, J. B. Park, Y. Lee, D. H. Kim, A. Ryu, J. Jeong, K. You, B. Ji, Z. Liu, Q. Huo, X. Feng, Y. Deng, Y. Xu, K.-I. Jang, J. Kim, Y. Zhang, R. Ghaffari, C. M. Rand, M. Schau, A. Hamvas, D. E. Weese-Mayer, Y. Huang, S. M. Lee, C. H. Lee, N. R. Shanbhag, A. S. Paller, S. Xu, J. A. Rogers, Binodal, wireless epidermal electronic systems with in-sensor analytics for neonatal intensive care. Science 363, eaau0780 (2019). 30819934 10.1126/science.aau0780 PMC6510306

[81] X. Liu, J. L. Berger, A. Ogirala, M. H. Mickle, A touchprobe method of operating an implantable RFID tag for orthopedic implant identification. IEEE Trans. Biomed. Circuits Syst. 7, 236–242 (2013). 23853323 10.1109/TBCAS.2012.2201258

[82] M. Kiani, K. Y. Kwon, F. Zhang, K. Oweiss, M. Ghovanloo, Evaluation of a closed loop inductive power transmission system on an awake behaving animal subject. Annual Int. Conf. IEEE Eng. Med. Biol. Soc. 2011, 7658–7661 (2011). 10.1109/IEMBS.2011.6091887 PMC3261583 22256112

[83] J. Charthad, M. J. Weber, T. C. Chang, A. Arbabian, A mm-sized implantable medical device (IMD) with ultrasonic power transfer and a hybrid bi-directional data link. IEEE J. Solid-State Circuits 50, 1741–1753 (2015).

[84] S. Ozeri, D. Shmilovitz, S. Singer, C. C. Wang, Ultrasonic transcutaneous energy transfer using a continuous wave 650 kHz Gaussian shaded transmitter. Ultrasonics 50, 666–674 (2010). 20219226 10.1016/j.ultras.2010.01.004

