# Supplementary material for: A flexible, stretchable system for simultaneous acoustic energy transfer and communication
Source: Sci Adv. 2021 Sep 29;7(40):eabg2507. doi: 10.1126/sciadv.abg2507 (PMC8480923; doi:10.1126/sciadv.abg2507)
Supplement: Supplementary file 1 — Supplementary Notes S1 to S10 Figs. S1 to S19 Table S1 References [file sciadv.abg2507_sm.pdf]

Science Advances
AAAS

Supplementary Materials for

A flexible, stretchable system for simultaneous acoustic energy transfer and communication

Peng Jin, Ji Fu, Fengle Wang, Yingchao Zhang, Peng Wang, Xin Liu, Yang Jiao, Hangfei Li, Ying Chen, Yinji Ma, Xue Feng*

*Corresponding author. Email: fengxue@tsinghua.edu.cn

Published 29 September 2021, *Sci. Adv.* **7**, eabg2507 (2021)
DOI: 10.1126/sciadv.abg2507

This PDF file includes:

Supplementary Notes S1 to S10
Figs. S1 to S19
Table S1
References

Supplementary Notes

Supplementary Note 1: Temperature measurement process from AECD in the acoustic communication experiment.

For the temperature sensor, the response relationship between temperature and output voltage can be described by the following equation from the chip datasheet, where T_M is the measured temperature and V_{TAO} is the temperature sensor's voltage output.

$$T_M = -0.193°C / mV \times V_{TAO} + 212.009°C, \quad \text{(S1)}$$

The AECD's ADC set up is that ADC reference voltage is set as 1.1V and resolution is set as 8-bit. For the temperature sensor, and the ADC measurement result can be calculated by the following equation.

$$ADC = \frac{V_{IN} \times 256}{V_{REF}}, \quad \text{(S2)}$$

where ADC, V_{IN} and V_{REF} is the acquired result (an integer number), the measured analog electrical signal (here $V_{IN} = V_{TAO}$) and the ADC reference voltage (here $V_{REF} = 1.1V$).

Take a temperature measurement calculation for example. When the environment temperature is about 26.2°C ($T_M = 26.2°C$),according to the thermoelectrical response, the temperature sensor would output an analog electrical signal ($V_{TAO} = 962.7mV = V_{IN}$), calculated based on S1. After ADC process, the MCU inside the AECD would get an integer number ($ADC = 224$), calculated based on S2. To minimize data cycle size, the obtained integer subtracts 200 to be used as the ultimate data (here $ultimateData = ADC - 200 = 24$). Next, through an IO port, according the ultimate data, the AECD would transmit an intermittent pulse signal (here contains 24 pulse number in one data cycle) to simulate the emissive ultrasonic transducer, making it to transmit specific ultrasound that carries the temperature data.

Supplementary Note 2: The mean relative error calculation process.

The mean relative error calculation is based on the following equation,

$$MRE = \frac{1}{N_{Sample}} \sum_{n=0}^{N_{Sample}} \left(\left| \frac{X_{meas} - X_{true}}{X_{true}} \right| \right), \quad \text{(S3)}$$

Where $X_{meas}, X_{true}, N_{Sample}, MRE$ are the measured value, the true value, total sample number and mean relative error calculation result.

Supplementary Note 3: Calculation of the SNR in the acoustic communication experiment.

In the acoustic communication, frequency analysis (Fig S8) have shown great SNR (Signal to Noise Ratio) for the received communication ultrasound. Here, the received signal is divided into two parts: signal part and noise part. In order to quantitative analysis the noise level, SNR is calculated based on the following formula.

$$SNR = 10\log_{10}\left(\frac{Signal \quad power}{Noise \quad power}\right), \tag{S4}$$

Where,

$$Signal \quad power = \frac{1}{N_{Signal}}\sum_{n=0}^{N_{Signal}}\left|X_{n,Signal}\right|^2, \tag{S5}$$

$$Noise \quad power = \frac{1}{N_{Noise}}\sum_{n=0}^{N_{Noise}}\left|X_{n,Noise}\right|^2, \tag{S6}$$

where, $X_{n,Signal}$, $X_{n,Noise}$, N_{Signal} and N_{Noise} is the signal sample value, the noise sample value, the signal sample number and the noise sample number.

Therefore, for the received communication ultrasound, the SNR is 16.8192.

Supplementary Note 4: Calculation of the acoustic power and energy efficiency.

Supplementary Figure S13 has explored the acoustic energy transfer power and efficiency. At that experiment, the consumed power Pin is the power applied on the transmitter ultrasound transducer, which is calculated by the following formula.

$$P_{in} = \frac{\int U_{in} I_{in} dt}{\Delta t}, \tag{S7}$$

where, U_{in} and I_{in} is the voltage and current of the transmitter ultrasound transducer.

The received power Pout is the power of the receiver system, which is calculated by the following formula.

$$P_{out} = \left(\frac{U_{eff}}{R}\right)^2 (R+r) \tag{S8}$$

$$U_{eff} = \frac{U_{out,peak-peak}}{2\sqrt{2}} \tag{S9}$$

$$R = r = 110\Omega \tag{S10}$$

where, U_{eff}, $U_{out,peak-peak}$, R and r is the voltage effective value and peak-peak value of the receiver ultrasound transducer, load resistance value in receiver system and internal resistance value of the receiver ultrasound transducer at 2MHz.

The energy transfer efficiency is calculated by the following formula.

$$\eta_{efficiency} = \frac{P_{out}}{P_{in}} \tag{S11}$$

Supplementary Note 5: Alignment between internal ultrasound transducers and external transducers.

Here is how we did the alignment. When implanting the AECD inside the tissue, we recorded how deep we put it in and estimated the positions of the internal transducers (internal receiver transducer and internal transmitting transducer), then we moved the external transducers around that estimated the positions meanwhile recording the response voltage of the external receiver transducer until the response got maximum.

Supplementary Note 6: Data bandwidth and transmission performance of the AECD based on the acoustic communication method.

Here, the maximum data bandwidth is at least up to 0.4kHz ($f_{data} = 407Hz$, Fig S8b). While, the frequency of the rabbit heart beat signal is about 38Hz ($f_{ECG} = 38Hz$, Fig S16). According to the Shannon's sampling theorem, in order to recover the measured signal, the sampling frequency should be at least upper then 2 times of signal frequency ($f_{sampling} \geq 2 f_{signal}$). Therefore, here, the AECD is capable of capturing and transmitting the rabbit's ECG signal ($f_{data} \gg 2 f_{ECG}$).

Supplementary Note 7: Illustration of the AECD's stretchability principle.

The function components' rigidity are much bigger then the encapsulation part and the ultrathin serpentine interconnections, so when applying deformation (for example stretching the system), the encapsulation part and ultrathin serpentine interconnections would be stretched, resulting in the AECD's being stretched, while during this process the rigid function components can keep almost undeformed (A detailed stretch and recover process could well demonstrated that, in Fig S14).

Supplementary Note 8: Analysis of the influence of layer materials variance on the ultrasonic energy transmission for the EETE.

According to acoustic theory, when the ultrasound travels across the interface between two material layers, the incident wave will be divided into reflected wave and transmitted wave. The acoustic energy ratio between the transmitted wave and the incident wave can be determined by the transmission coefficient of acoustic intensity τ_I. In vertical incidence situation, τ_I can be calculated by the following equation (S12):

$$\tau_I = \frac{I_t}{I_i} = \frac{4Z_1Z_2}{\left(Z_1 + Z_2\right)^2} \qquad \text{(S12)}$$

Where, I_t, I_i denotes the acoustic intensity of transmitted wave and incident wave; Z_1, Z_2 denotes the acoustic impedance of the layers traveled by transmitted wave and incident wave.

Here, for the structure of EETE, there exist two acoustic interfaces: Ecoflex-Water interface and Ecoflex-Perspex interface. The structure illustration and material acoustic impedance value are shown in Supplementary Figure S9.

According to the equation S12, we have individually calculated the transmission coefficient of acoustic intensity τ_I for these two interfaces. The calculated result is shown in the Supplementary Figure S17. According to the result (Fig. S17c), the transmission loss for ultrasound traveling across the interface is mild, that is about 1.61% for Ecoflex-Water interface and 6.52% for Ecoflex-Perspex interface.

Supplementary Note 9: Analysis of the influence of receiving angle on the ultrasonic energy transmission.

We have explored the energy receiving efficiency vs the receiving angle through simulation (Fig S18). In this simulation, we have characterized the received energy by $\sum P_{\mathrm{i}}^2$ (P_{i} is the acoustic pressure in the received plate). According to the result, as shown in the Supplementary Figure S18, when the deflection angle is within 10 degree, the energy efficiency drops rapidly (from 100% $\alpha = 0^\circ$ to 76% $\alpha = 10^\circ$) and then the drop-speed slows down (from 76% $\alpha = 10^\circ$ to 63% $\alpha = 30^\circ$).

Supplementary Note 10: Acoustic transmission performance comparing between the EETE and a single PZT plate.

Here, we have compared the acoustic transmission performance between the EETE and a single PZT plate through simulation, as shown in the Supplementary Figure S19. In the acoustic pressure field simulation (acoustic pressure is directly related with the acoustic energy), in the sake of setting equal excitation condition and environmental condition, here we set the transmitter length of EETE's single element as ds, and set the transmitter length of a single PZT plate as $5ds$. Moreover, in order to characterizing

the focus effect, for both two simulation models, we considering the receiver point at the same depth place (at the focus point). In addition, for both two simulation models, the initial pressure excitation is all set as 10 Pa. According to the result (Supplementary Figure S19c), the received acoustic pressure by the EETE is about 7.25 times of by a single PZT plate.

Supplementary Figures

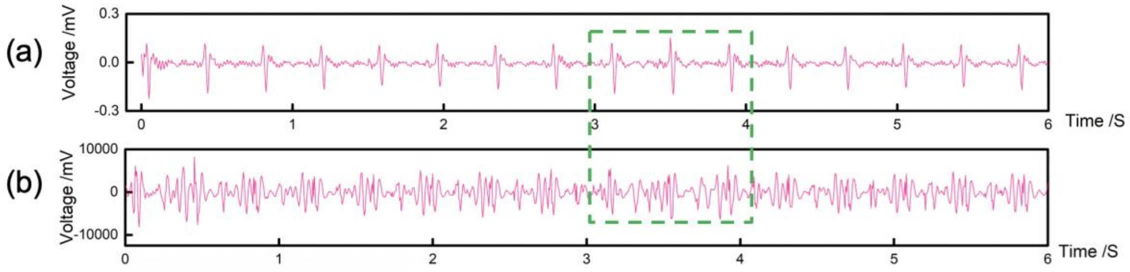

Supplementary Figure S1 | Additional instructions for cardiac pacing experiments

(**a**) Recording Rabbit's normal ECG. (**b**) Recording PVDF sensor's signal when attached on rabbit's heart.

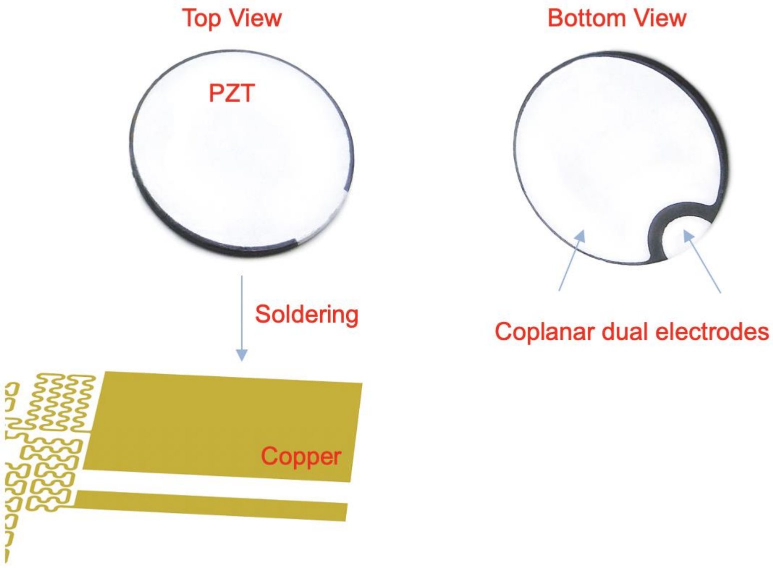

Supplementary Figure S2 | Illustration, shows PZTs and its' soldering connections.

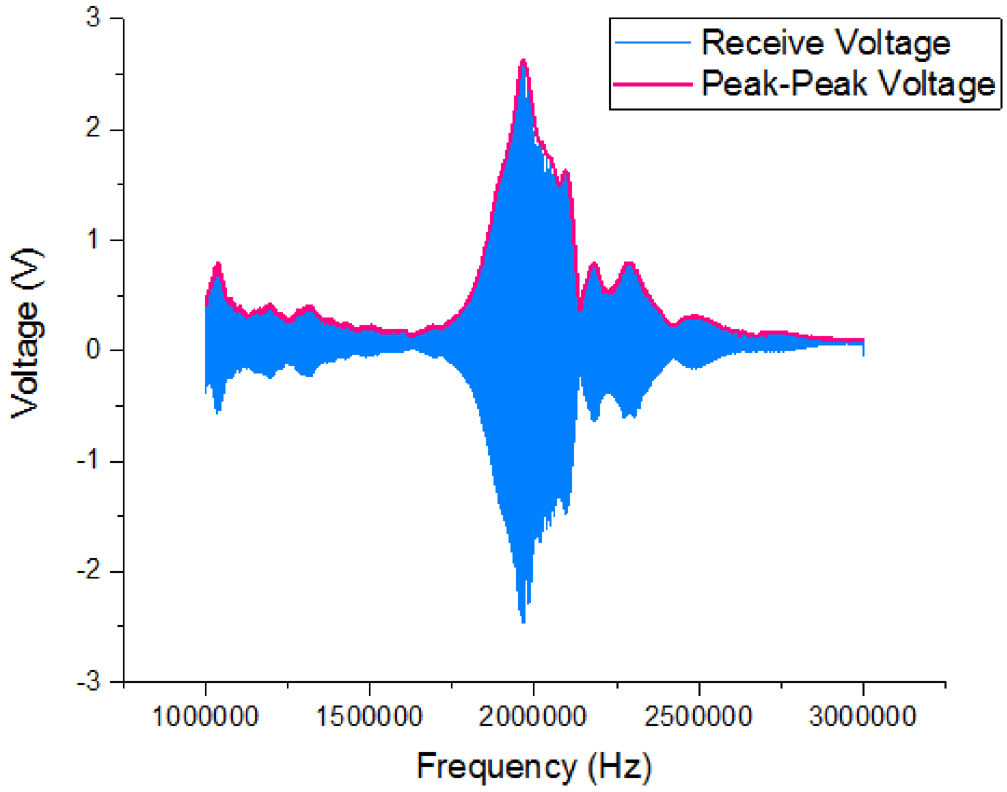

Supplementary Figure S3 | Resonant frequency testing of the cured PZTs, using a swept-frequency signal to simulated an ultrasonic probe and simultaneously recording the voltage response of the cured PZTs.

(a)

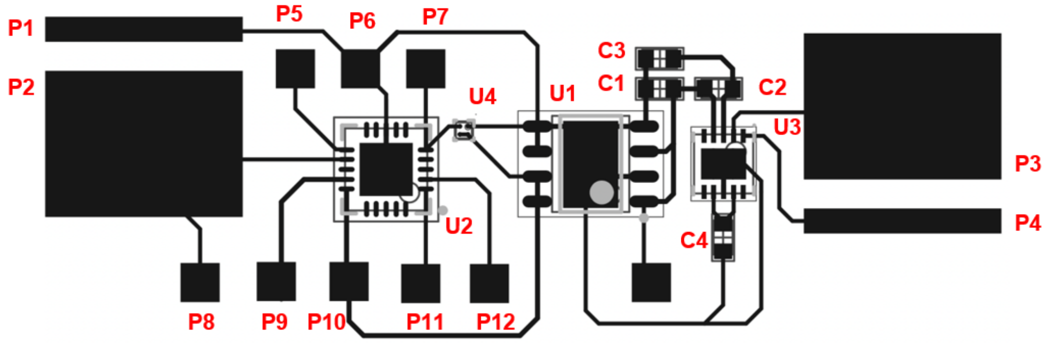

(b)

Designator	Component	Value or Product Number
C1、C2	Capacitance	1uF
C3	Capacitance	10uF
C4	Capacitance	4.7uF
U1	Voltage Reference	MCP1501
U2	Microcontroller	ATtiny85V
U3	Rectifier	LTC3588
U4	Temperature Sensor	LMT70YFQR
P1-P12	Copper pad	-

Supplementary Figure S4 | Layout and component information of the AECD. (a) Electrical layout diagrams of AECD. (b) Components information used in AECD.

(a)

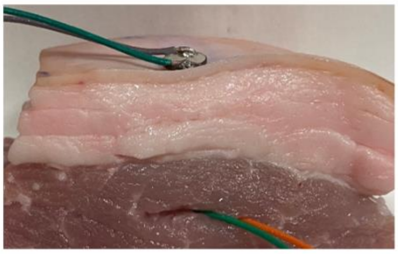

(b)

Transmit Deepness (mm)	5	10	20	30	40	50	50
Response Peak-Peak Voltage (mV)	4256	3892	3080	2408	2156	1876	5275
Response Voltage Root Mean Square (mV)	1505	1376	1089	851	762	663	1865
Excitation Peak-Peak Voltage (mV)	18000	17360	18540	19440	19100	19300	37100
Media Layer	Fat	Fat	Fat	Fat / Muscle	Fat / Muscle	Fat / Muscle	Fat

Supplementary Figure S5 | Ultrasonic energy transfer test cross biological tissue with depth (ultrasound frequency is 2MHz). (a) Optical picture of the experiment setup, using fat and muscle as transmission media. (b) The ultrasonic energy transfer efficiency, with different transfer depth and transmission media. ("Photo Credit: Peng Jin, Tsinghua University")

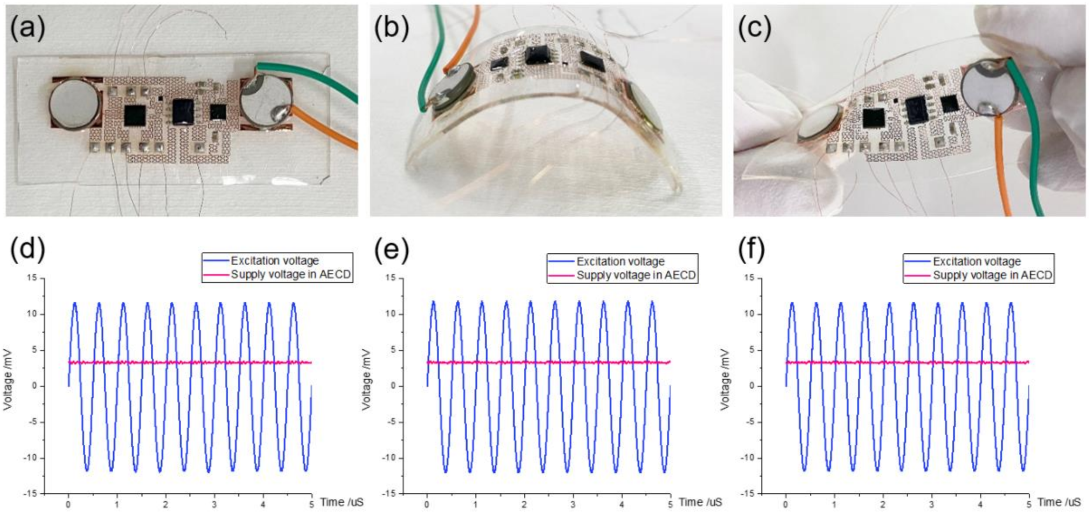

Supplementary Figure S6 | Electrical robustness of AECD under different deformation configuration (ultrasound frequency is 2MHz). Optical picture of the AECD, receiving ultrasound and converting it to electrical energy under different deformation configuration (a) in flatten state, (b) in bent state and (c) in twisted state. The excitation voltage versus the supply voltage in AECD when it’s under different deformation configuration (d) in flatten state, (e) in bent state and (f) in twisted state. ("Photo Credit: Peng Jin, Tsinghua University")

(a)

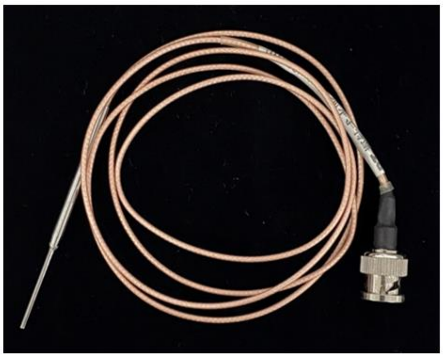

(b)

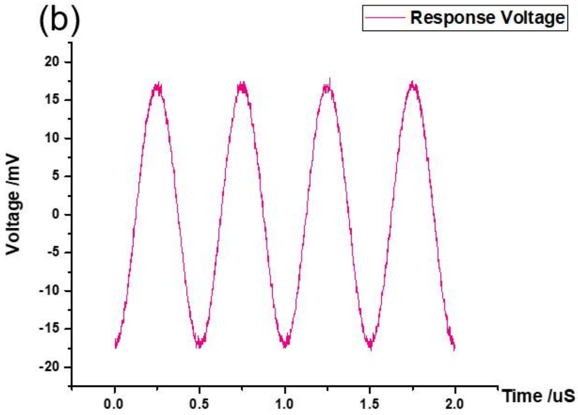

(c)

Frequency(MHz)	Conversion coefficient(uV/Pa)
1.8	0.19
1.9	0.22
**2.0**	**0.21**
2.1	0.22
2.2	0.16

Supplementary Figure S7 | Maximum sound pressure experiment. (a) Optical picture of the hydrophone, used for measuring the sound pressure. (b) Maximum response voltage of the hydrophone, measuring at the bottom center point of EETE when it’s the focus point of EETE, and the ultrasound frequency adopt here is 2MHz. (c) Table of the hydrophone’s conversion coefficient. ("Photo Credit: Peng Jin, Tsinghua University")

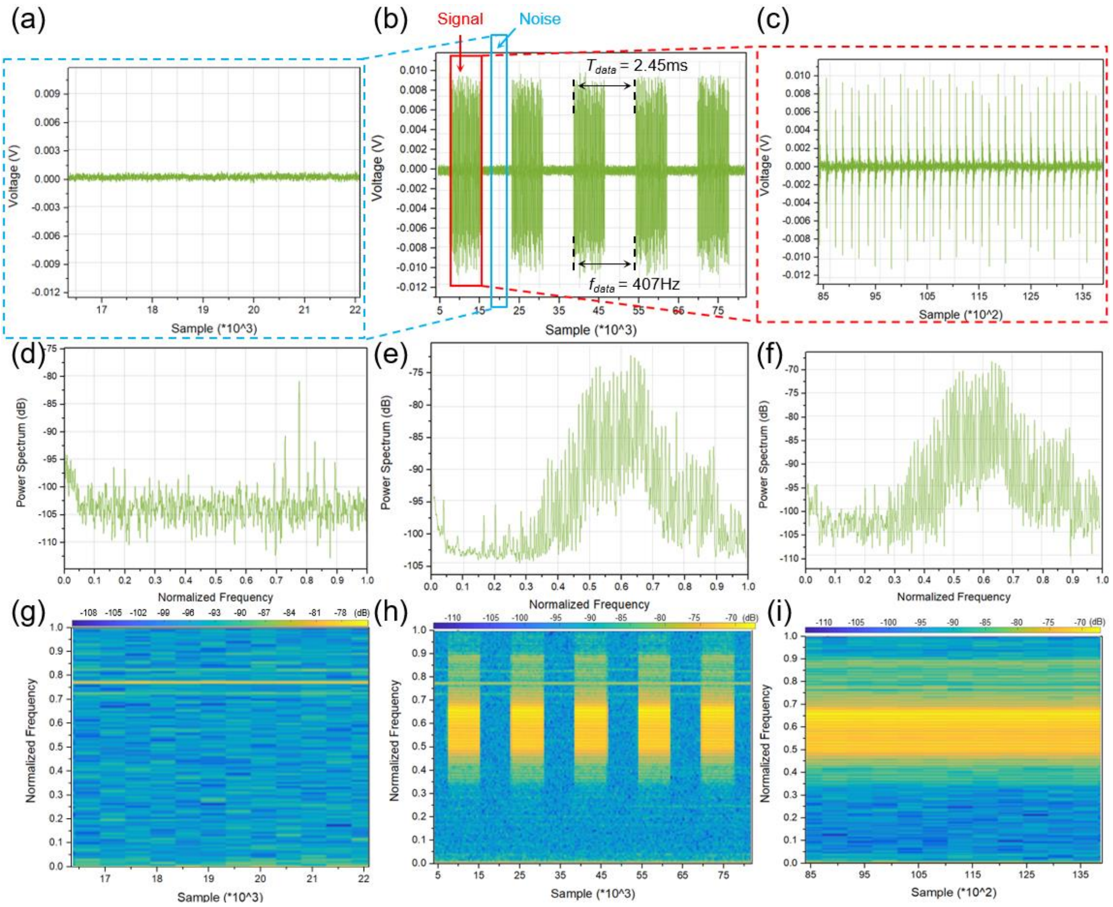

Supplementary Figure S8 | Frequency analysis for the ultrasound signal in the acoustic communication experiment. (b) The ultrasound signal in time-domain, transmitted by the AECD in the acoustic communication experiment, (a) the zoomed ultrasound signal in time-domain and (c) the zoomed noise signal in time-domain. (e) Power spectrum of the ultrasound signal, transmitted by the AECD in the acoustic communication experiment, (d) power spectrum of the zoomed ultrasound signal and (f) power spectrum of the zoomed noise signal. (h) Spectrogram of the ultrasound signal, transmitted by the AECD in the acoustic communication experiment, (g) spectrogram of the zoomed ultrasound signal and (i) spectrogram of the zoomed noise signal.

(a)

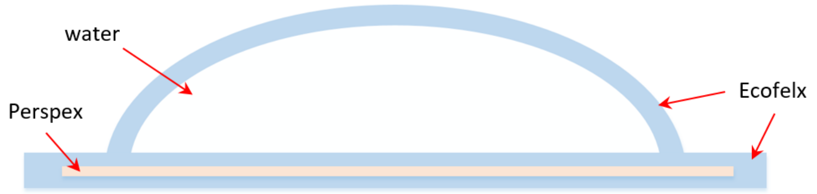

(b)

Material	Density (kg/m^3)	Wave Velocity (m/s)	Acoustic Impedance (MRayl)
Ecoflex	1154	1658	1.91
Water[72]	997	1498	1.48
Perspex[72]	1180	2730	3.22

Supplementary Figure S9 | Acoustic impedance analysis of EETE (a) Structure illustration of EETE. (b) Acoustic property of different layer materials of EETE. (Data of Ecoflex is determined by experiment, while data of Water and Perspex is referred from scholar Rathod (*76*))

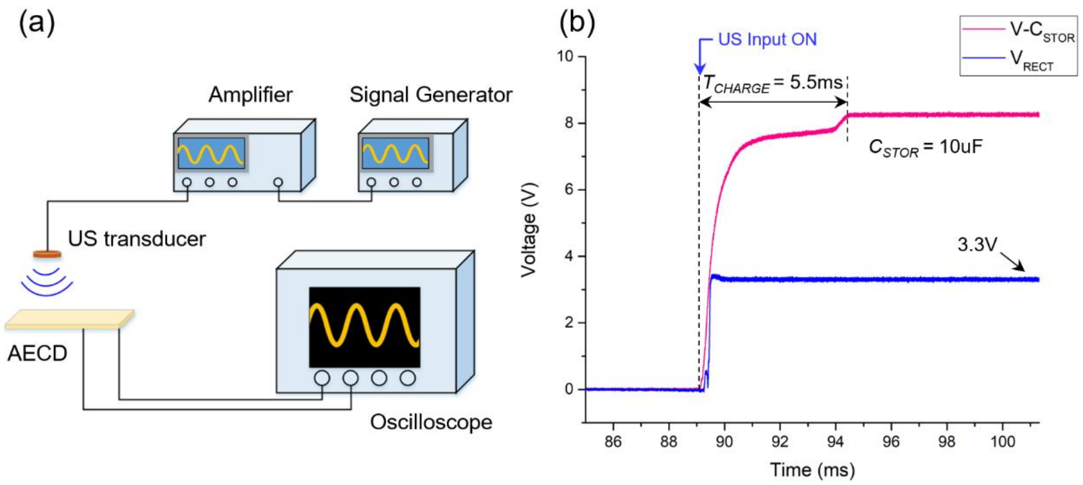

Supplementary Figure S10 | Charging rate characterization of the AECD. (a) Illustration of the experiment setup. (b) Measured power recovery signal of the AECD during charging process.

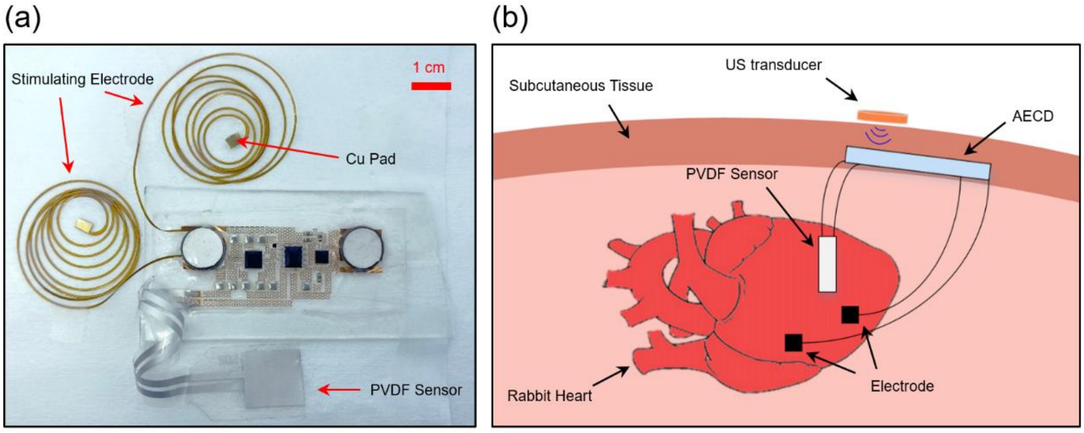

Supplementary Figure S11 | Structure illustration of the AECD used in the in vivo animal experiment. (a) Connection illustration of the stimulating electrode and the PVDF sensor on the AECD. (b) Illustration of the device placement in the in vivo animal experiment. ("Photo Credit: Peng Jin, Tsinghua University")

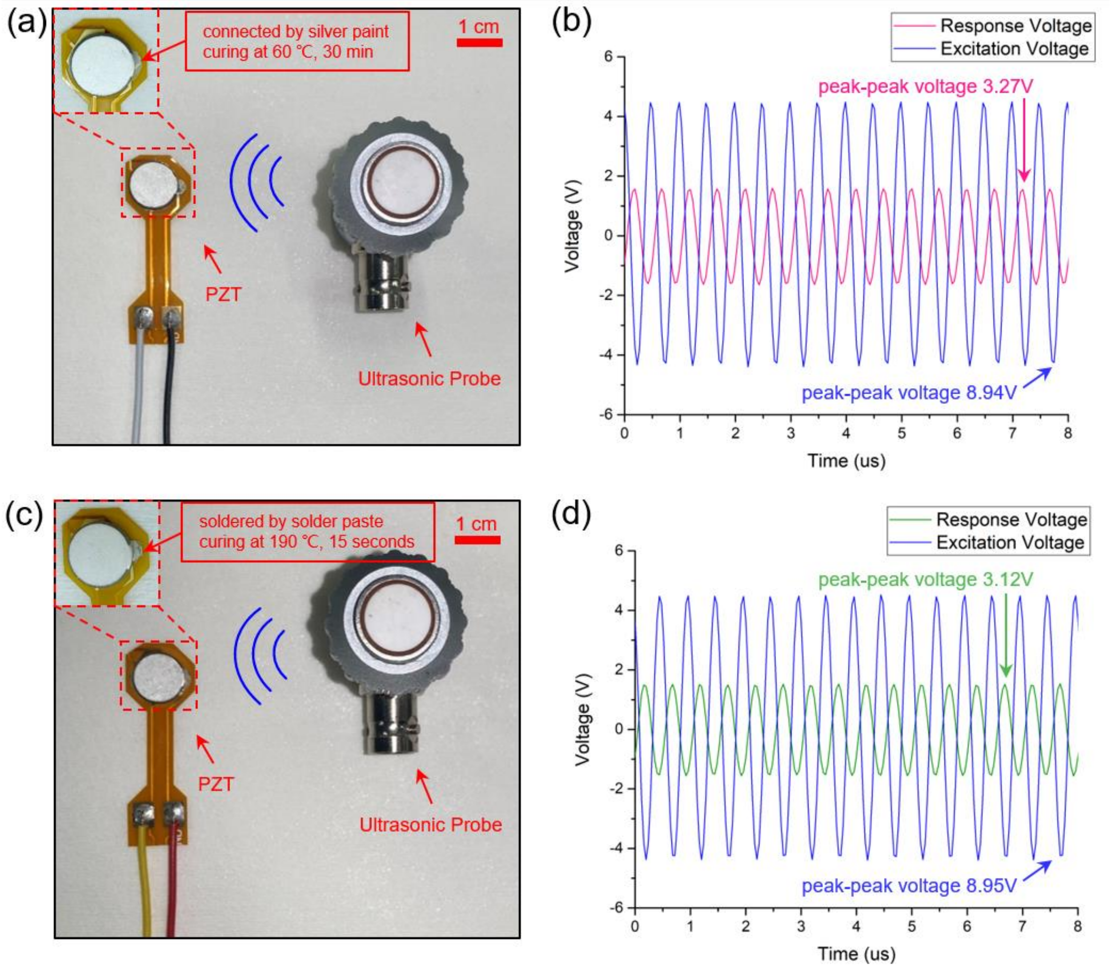

Supplementary Figure S12 | High-temperature reflow process influence on the acoustic performance of the PZT. Optical images of the ultrasound emitter (an ultrasonic probe) and the ultrasound receiver (a PZT plate), that (a) the PZT plate is connected by silver paint curing at 60℃ for 30 min and (c) the PZT plate is soldered by solder paste curing at 190℃ for 15 seconds. Acoustoelectric response of PZT plate, that (a) the receiver PZT plate is connected by silver paint and (b) the receiver PZT plate is soldered by solder paste, with the same excitation applying on the ultrasound emitter (an ultrasonic probe). ("Photo Credit: Peng Jin, Tsinghua University")

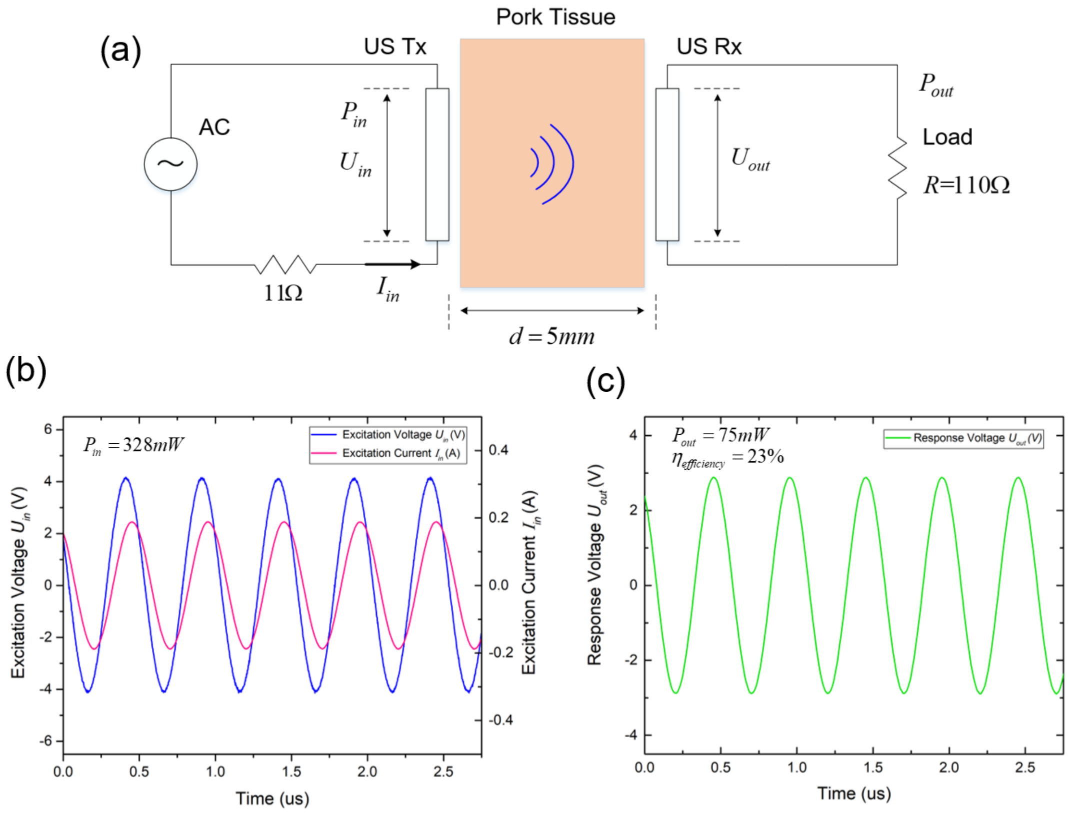

Supplementary Figure S13 | Power and efficiency characterization of the acoustic energy transfer method cross pork tissue. (a) Schematic diagram of electrical connection, that the receiver ultrasound transducer is connected in series with a load resistance at 110 Ohm, which is close to the matched transducer's resistance at 2MHz. (b) Measured electrical waveforms of the excitation voltage U_{in} and current I_{in} for calculating the consumed power P_{in}. (c) Measured electrical waveform of the response voltage of the receiver ultrasound transducer for calculating the received power P_{out}.

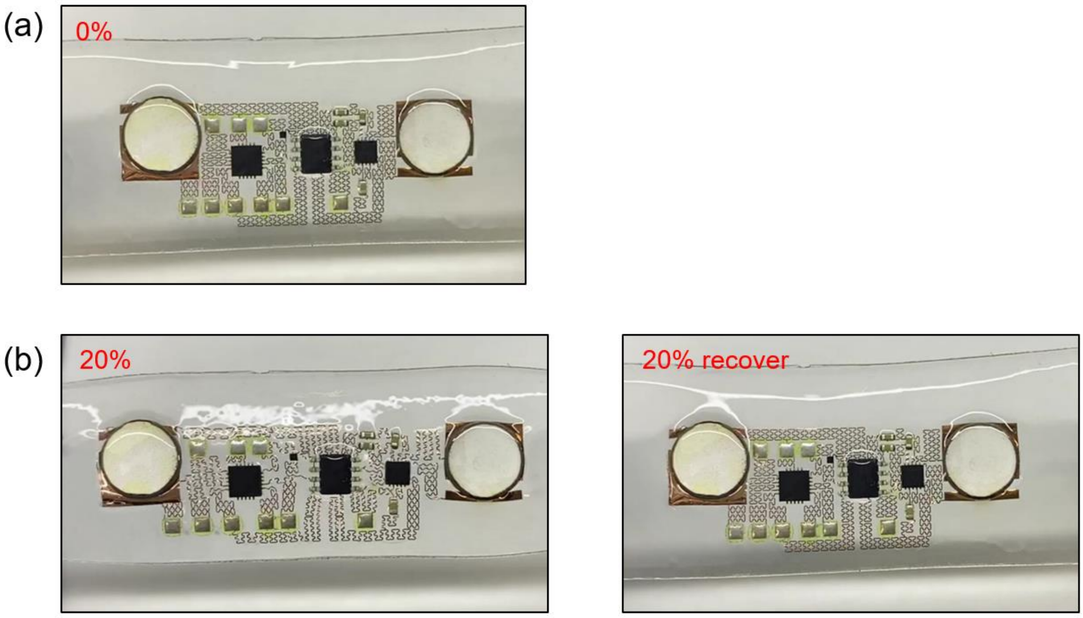

Supplementary Figure S14: Images of the uniaxial tensile test. (a) The 0% original state. (b) The 20% tensile state of the entire device and its recovery. ("Photo Credit: Peng Jin, Tsinghua University")

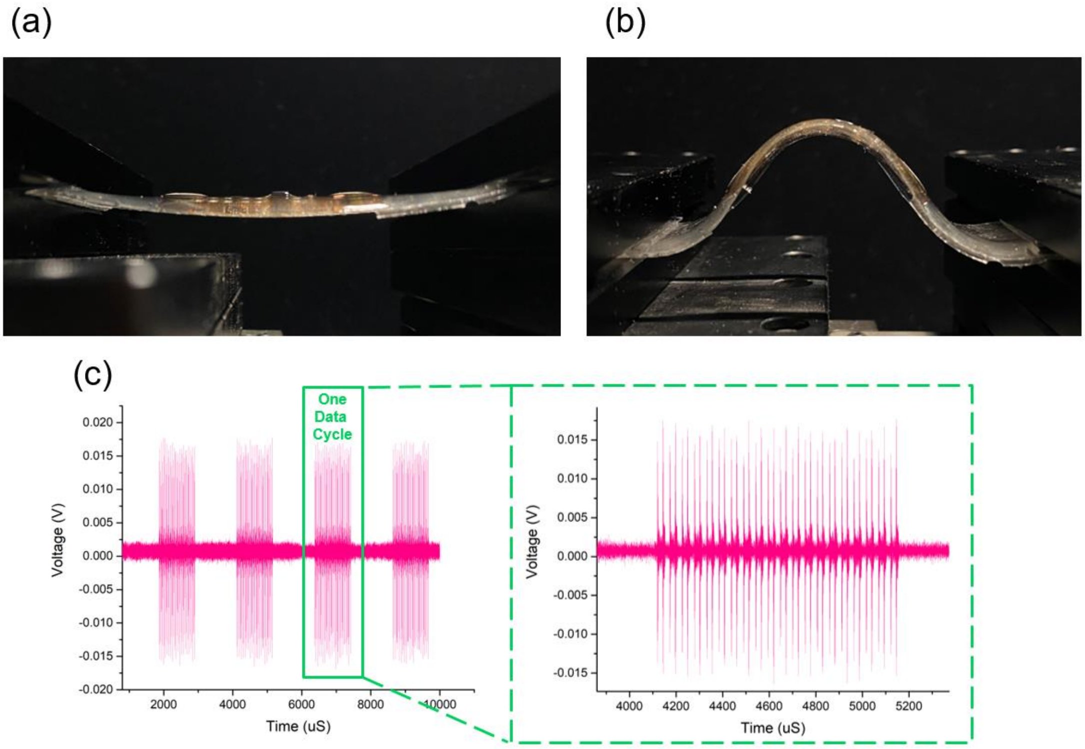

Supplementary Figure S15: Repeat mechanical testing. (a) Optical image of the device without load. (b) Optical image of the device under bending load configuration. (c) wireless acoustic communication result (pulse number at 220, measured temperature at 29℃) performed by the AECD after repeat bending load (500 times load, bending angle at 80 degree, load speed at 1 circle/second). ("Photo Credit: Peng Jin, Tsinghua University")

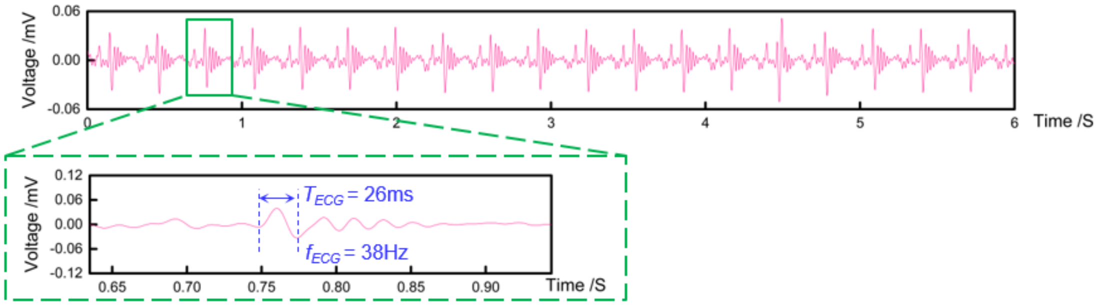

Supplementary Figure S16: Frequency analysis of the rabbit's ECG signal.

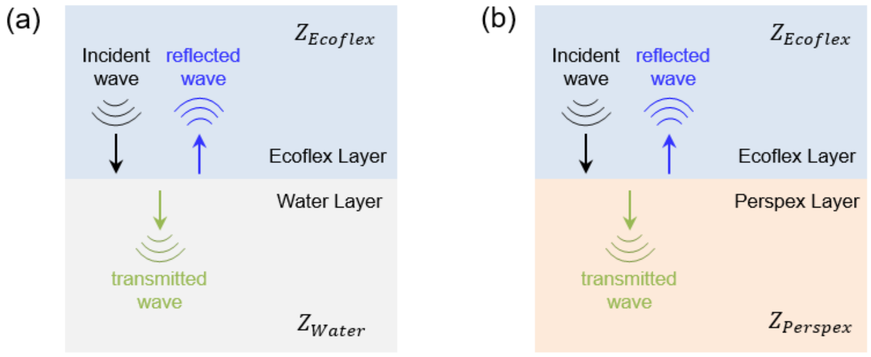

(c)

Acoustic impedance		Transmission coefficient of acoustic intensity	
$Z_{Ecoflex}$	1.91 MRayl	$\tau_{I\_Ecoflex\_Water} = \frac{4Z_{Water}Z_{Ecoflex}}{(Z_{Water} + Z_{Ecoflex})^2}$	0.9839
Z_{Water}	1.48 MRayl		
$Z_{Perspex}$	3.22 MRayl	$\tau_{I\_Ecoflex\_Perspex} = \frac{4Z_{Perspex}Z_{Ecoflex}}{(Z_{Perspex} + Z_{Ecoflex})^2}$	0.9348

Supplementary Figure S17: The influence of layer materials variance on the ultrasonic energy transmission. The acoustic reflection and transmission illustration on (a) Ecoflex-Water interface and (b) Ecoflex-Perspex interface. (c) A table shows the transmission coefficient of acoustic intensity for Ecoflex-Water interface and Ecoflex-Perspex interface.

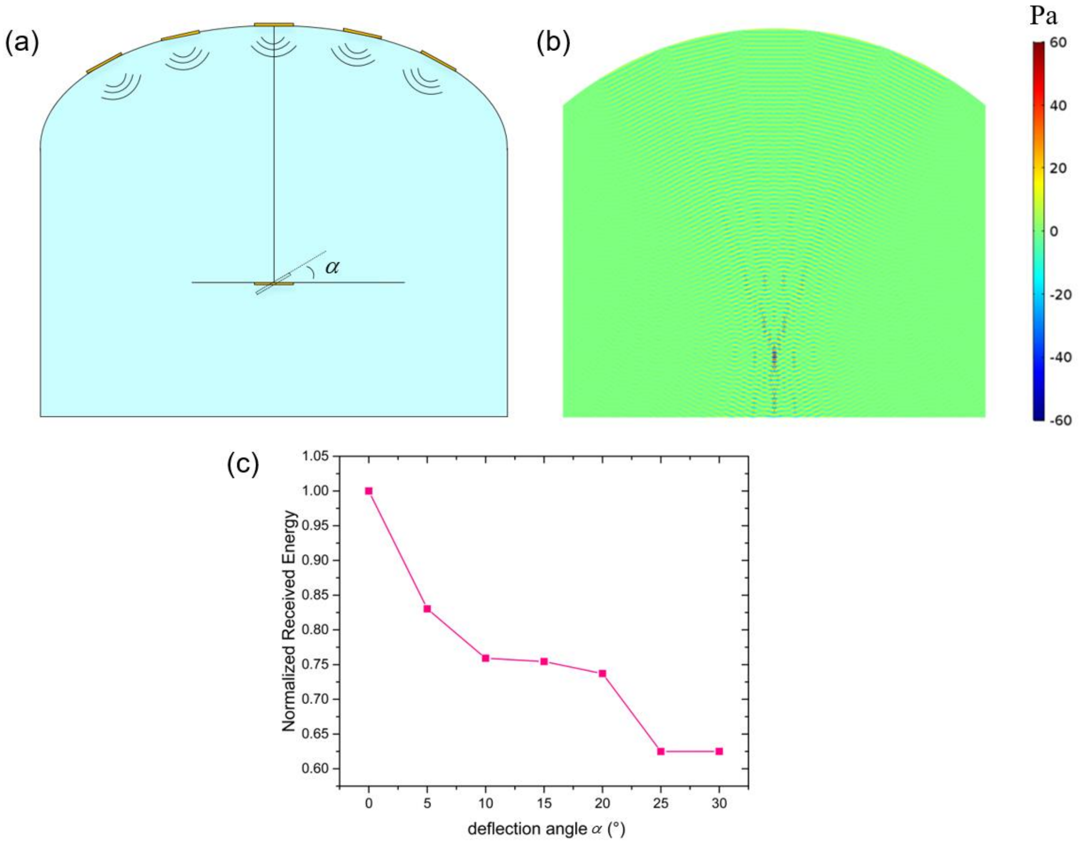

Supplementary Figure S18: The influence of receiving angle on the ultrasonic energy transmission. (a) Illustration of the geometric relations. (b) Acoustic pressure simulation result for the EETE. (c) Received energy vs different deflection angle α, that the result is normalized by the maximum received energy(α=0).

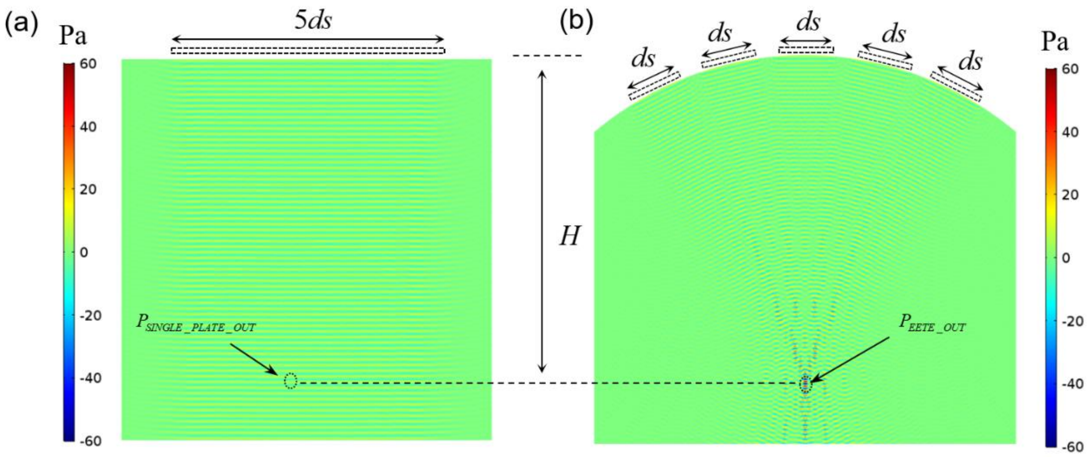

(c)

$\xi = \frac{P_{EETE\_OUT}}{P_{SINGLE\_PLATE\_OUT}}$	$P_{SINGLE\_PLATE\_OUT}$	P_{EETE_OUT}	ds	H
7.25	8 Pa	58 Pa	10mm	54mm

Supplementary Figure S19: Acoustic transmission performance comparing between the EETE and a single PZT plate. (a) Acoustic pressure field simulation result for a single PZT plate. (b) Acoustic pressure field simulation result for the EETE. (c) A table, showing the acoustic transmission comparing between the EETE and a single PZT plate.

Supplementary Table

Method	Year/Ref	Device dimension	Power intensity (mW/cm^{-2})	Efficiency (%)	Frequency (MHz)	Distance (mm)	stretchable
Acoustic (APT)	2021/ Our device	6 × 2.5 cm	95	23	2	50	Yes
	2020/(*49*)	0.31 × 0.19 × 0.08 cm	56	20.6	1.85	18	No
	2015/(*82*)	0.4 × 0.78 cm	36	-	30	60	No
	2010/(*83*)	1.4 cm^2 (transducer size)	71	39	0.65	5	No
Inductive coupling (IPT)	2019/(*71*)	3 × 3 × 0.3 cm	0.42	-	13.56	-	Yes
	2013/(*80*)	3 × 1 cm	0.32	-	13.56	24	No
	2012/(*76*)	70 cm^2 (transducer size)	113	90	13.56	71	No
	2011/(*81*)	1.2 cm^2 (transducer size)	22.5	4.82	13.56	7	No
Radio frequency (RF)	2019/(*79*)	4.77 × 2.26 cm	0.07	-	128	29	Yes
	2017/(*77*)	1.16 × 1.59 cm	0.27	0.03	1200	58	No
	2014/(*15*)	0.06 cm^2 (transducer size)	5.97	0.08	1600	20	No
	2011/(*78*)	0.78 cm^2 (transducer size)	0.0029	1.73	2400	15	No

Supplementary Table S1: Comparison of our device with other existing system based on different wireless powering mechanisms.

REFERENCES AND NOTES

1. E. H. Ledet, D. D'Lima, P. Westerhoff, J. A. Szivek, R. A. Wachs, G. Bergmann, Implantable sensor technology: From research to clinical practice. *J. Am. Acad. Orthop. Surg.* **20**, 383–392 (2012).

2. J. Lueke. W. A. Moussa, MEMS-based power generation techniques for implantable biosensing applications. *Sensors* **11**, 1433–1460 (2011).

3. P. Gerrish, E. Herrmann, L. Tyler, K. Walsh. Challenges and constraints in designing implantable medical ICs. *IEEE Trans. Device Mater. Reliab.* **5**, 435–444 (2005).

4. P. R. Troyk, G. A. DeMichele, D. A. Kerns, R. F. Weir, in *2007 Annual International Conference of the IEEE Engineering in Medicine and Biology Society, Vols 1–16 Proceedings of Annual International Conference of the IEEE Engineering in Medicine and Biology Society* (IEEE, 2007), pp. 1730–1733.

5. K. D. Wise, D. J. Anderson, J. F. Hetke, D. R. Kipke, K. Najafi, Wireless implantable microsystems: High-density electronic interfaces to the nervous system. *Proc. IEEE* **92**, 76–97 (2004).

6. F. V. Y. Tjong, V. Y. Reddy, Permanent leadless cardiac pacemaker therapy: A comprehensive review. *Circulation* **135**, 1458–1470 (2017).

7. A. Guiseppi-Elie, S. Brahim, G. Slaughter, K. R. Ward, Design of a subcutaneous implantable biochip for monitoring of glucose and lactate. *IEEE Sens. J.* **5**, 345–355 (2005).

8. D. C. Bock, A. C. Marschilok, K. J. Takeuchi, E. S. Takeuchi, Batteries used to power implantable biomedical devices. *Electrochim. Acta* **84**, 155–164 (2012).

9. F. Akhtar, M. H. Rehmani, Energy replenishment using renewable and traditional energy resources for sustainable wireless sensor networks: A review. *Renew. Sustain. Energy Rev.* **45**, 769–784 (2015).

10. S. Kerzenmacher, J. Ducree, R. Zengerle, F. von Stetten, Energy harvesting by implantable abiotically catalyzed glucose fuel cells. *J. Power Sources* **182**, 1–17 (2008).

11. M. A. Hannan, S. Mutashar, S. A. Samad, A. Hussain, Energy harvesting for the implantable biomedical devices: Issues and challenges. *Biomed. Eng. Online* **13**, 79 (2014).

12. A. Ben Amar, A. B. Kouki, H. Cao Power approaches for implantable medical devices. *Sensors* **15**, 28889–28914 (2015).

13. A. Kim, M. Ochoa, R. Rahimi, B. Ziaie, New and emerging energy sources for implantable wireless microdevices. *IEEE Access* **3**, 89–98 (2015).

14. S. O'Driscoll, A. S. Y. Poon, T. H. Meng, Wireless power transmission for implantable medical devices. U.S. Patent 08634928 (2014).

15. T. Stuart, L. Cai, A. Burton, P. Gutruf, Wireless and battery-free platforms for collection of biosignals. *Biosens. Bioelectron.* **178**, 113007 (2021).

16. A. D. Mickle, S. M. Won, K. N. Noh, J. Yoon, K. W. Meacham, Y. Xue, L. A. McIlvried, B. A. Copits, V. K. Samineni, K. E. Crawford, D. H. Kim, P. Srivastava, B. H. Kim, S. Min, Y. Shiuan, Y. Yun, M. A. Payne, J. Zhang, H. Jang, Y. Li, H. Henry Lai, Y. Huang, S.-I. Park, R. W. Gereau IV, J. A. Rogers, A wireless closed-loop system for optogenetic peripheral neuromodulation. *Nature* **565**, 361–365 (2019).

17. J. S. Ho, A. J. Yeh, E. Neofytou, S. Kim, Y. Tanabe, B. Patlolla, R. E. Beygui, A. S. Y. Poon, Wireless power transfer to deep-tissue microimplants. *Proc. Natl. Acad. Sci. U.S.A.* **111**, 7974–7979 (2014).

18. D. K. Piech, B. C. Johnson, K. Shen, M. M. Ghanbari, K. Y. Li, R. M. Neely, J. E. Kay, J. M. Carmena, M. M. Maharbiz, R. Muller, A wireless millimetre-scale implantable neural stimulator with ultrasonically powered bidirectional communication. *Nat. Biomed. Eng.* **4**, 207–222 (2020).

19. S. Lee, A. J. Cortese, A. P. Gandhi, E. R. Agger, P. L. McEuen, A. C. Molnar, A 250µm x 57µm microscale opto-electronically transduced electrodes (MOTEs) for neural recording. *IEEE Trans. Biomed. Circuits Syst.* **12**, 1256–1266 (2018b).

20. W. Xiaojuan, L. Jing, Power sources and electrical recharging strategies for implantable medical devices. *Front. Energy* **2**, 1–13 (2008).

21. Y. Wu, Q. Chen, X. Ren, Z. Zhang, Efficiency optimization based parameter design method for the capacitive power transfer system. *IEEE Trans. Power Electron.* **36**, 8774–8785 (2021).

22. G. E. Santagati, T. Melodia, Experimental evaluation of impulsive ultrasonic intra-body communications for implantable biomedical devices. *IEEE Trans. Mob. Comput.* **16**, 367–380 (2017).

23. M. Chitre, S. Shahabudeen, L. Freitag, M. Stojanovic, I. Mts, in *Oceans 2008* (IEEE , 2008), pp. 1–10.

24. T. Dahl, J. L. Ealo, H. J. Bang, S. Holm. P. Khuri-Yakub, Applications of airborne ultrasound in human-computer interaction. *Ultrasonics* **54**, 1912–1921 (2014).

25. F. I. Thurston, H. E. Melton, Biomedical ultrasonics, in *IEEE Transactions on Industrial Electronics and Control Instrumentation* (IEEE, 1970), pp. 167–172.

26. W. Pang, X. Cheng, H. Zhao, X. Guo, Z. Ji, G. Li, Y. Liang, Z. Xue, H. Song, F. Zhang, Z. Xu, L. Sang, W. Huang, T. Li, Y. H. Zhang. Electro-mechanically controlled assembly of reconfigurable 3D mesostructures and electronic devices based on dielectric elastomer platforms. *Natl. Sci. Rev.* **7**, 342–354 (2020).

27. J. Song, X. Feng, Y. Huang, Mechanics and thermal management of stretchable inorganic electronics. *Natl. Sci. Rev.* **3**, 128–143 (2016).

28. M. Cai, S. Nie, Y. Du, C. Wang, J. Song, Soft elastomers with programmable stiffness as strain-isolating substrates for stretchable electronics. *ACS Appl. Mater. Interfaces* **11**, 14340–14346 (2019).

29. J. Zhao, Y. Zhang, X. Li, M. Shi, An improved design of the substrate of stretchable gallium arsenide photovoltaics. *J. Appl. Mech.* **86**, 031009 (2019).

30. S. Yin, Y. Su, A traction-free model for the tensile stiffness and bending stiffness of laminated ribbons of flexible electronics. *J. Appl. Mech.* **86**, 051011 (2019).

31. Y. Zhang, N. Zheng, Y. Cao, F. Wang, P. Wang, Y. Ma, B. Lu, G. Hou, Z. Fang, Z. Liang, M. Yue, Y. Li, Y. Chen, J. Fu, J. Wu, T. Xie, X. Feng, Climbing-inspired twining electrodes using shape memory for peripheral nerve stimulation and recording. *Sci. Adv.* **5**, eaaw1066 (2019).

32. H. Hu, X. Zhu, C. Wang, L. Zhang, X. Li, S. Lee, Z. Huang, R. Chen, Z. Chen, C. Wang, Y. Gu, Y. Chen, Y. Lei, T. Zhang, N. H. Kim, Y. Guo, Y. Teng, W. Zhou, Y. Li, A. Nomoto, S. Sternini, Q. Zhou, M. Pharr, F. L. di Scalea, S. Xu, Stretchable ultrasonic transducer arrays for three-dimensional imaging on complex surfaces. *Sci. Adv.* **4**, eaar3979 (2018).

33. J. Koo, S. B. Kim, Y. S. Choi, Z. Xie, A. J. Bandodkar, J. Khalifeh, Y. Yan, H. Kim, M. K. Pezhouh, K. Doty, G. Lee, Y.-Y. Chen, S. M. Lee, D. D'Andrea, K. Jung, K. H. Lee, K. Li, S. Jo, H. Wang, J.-H. Kim, J. Kim, S.-G. Choi, W. J. Jang, Y. S. Oh, I. Park, S. S. Kwak, J.-H. Park, D. Hong, X. Feng, C.-H. Lee, A. Banks, C. Leal, H. M. Lee, Y. Huang, C. K. Franz, W. Z. Ray, M. M. Ewan, S.-K. Kang, J. A. Rogers, Wirelessly controlled, bioresorbable drug delivery device with active valves that exploit electrochemically triggered crevice corrosion. *Sci. Adv.* **6**, eabb1093 (2020).

34. H. Jeong, J. A. Rogers, S. Xu, Continuous on-body sensing for the COVID-19 pandemic: Gaps and opportunities. *Sci. Adv.* **6**, eabd4794 (2020).

35. D.-H. Kim, R. Ghaffari, N. Lu, J. A. Rogers. Flexible and stretchable electronics for biointegrated devices. *Annu. Rev. Biomed. Eng.* **14**, 113–128 (2012).

36. S. Choi, H. Lee, R. Ghaffari, T. Hyeon, D.-H. Kim, Recent advances in flexible and stretchable bio-electronic devices integrated with nanomaterials. *Adv. Mater.* **28**, 4203–4218 (2016).

37. N. Lu, D.-H. Kim. Flexible and stretchable electronics paving the way for soft robotics. *Soft Robot.* **1**, 53–62 (2014).

38. C. Choi, J. Leem, M. S. Kim, A. Taqieddin, C. Cho, K. W. Cho, G. J. Lee, H. Seung, H. J. Bae, Y. M. Song, T. Hyeon, N. R. Aluru, S. W. Nam, D.-H. Kim, Curved neuromorphic image sensor array using a MoS_2-organic heterostructure inspired by the human visual recognition system. *Nat. Commun.* **11**, 5934 (2020).

39. G. D. Cha, T. Kang, S. Baik, D. Kim, S. H. Choi, T. Hyeon, D.-H. Kim, Advances in drug delivery technology for the treatment of glioblastoma multiforme. *J. Control. Release* **328**, 350–367 (2020).

40. K. W. Cho, W. H. Lee, B.-S. Kim, D.-H. Kim, Sensors in heart-on-a-chip: A review on recent progress. *Talanta* **219**, 121269 (2020).

41. S. Yao, A. Myers, A. Malhotra, F. Lin, A. Bozkurt, J. F. Muth, Y. Zhu, A wearable hydration sensor with conformal nanowire electrodes. *Adv. Healthc. Mater.* **6**, 1601159 (2017).

42. S. Yao, P. Swetha, Y. Zhu, Nanomaterial-enabled wearable sensors for healthcare. *Adv. Healthc. Mater.* **7**, 1700889 (2018).

43. S. Niu, N. Matsuhisa, L. Beker, J. Li, S. Wang, J. Wang, Y. Jiang, X. Yan, Y. Yun, W. Burnett, A. S. Y. Poon, J. B.-H. Tok, X. Chen, Z. Bao, A wireless body area sensor network based on stretchable passive tags. *Nat. Electron.* **2**, 361–368 (2019).

44. M. L. Scarpello, D. Kurup, H. Rogier, D. Vande Ginste, F. Axisa, J. Vanfleteren, W. Joseph, L. Martens, G. Vermeeren, Design of an implantable slot dipole conformal flexible antenna for biomedical applications. *IEEE Trans. Antennas Propag.* **59**, 3556–3564 (2011).

45. Z. Xie, R. Avila, Y. Huang, J. A. Rogers, Flexible and stretchable antennas for biointegrated electronics. *Adv. Mater.* **32**, 1902767 2019.

46. Z. Xie, B. Ji, Q. Huo, Mechanics design of stretchable near field communication antenna with serpentine wires. *J. Appl. Mech.* **85**, 045001 (2018).

47. K.-G. Lim, S. Ahn, Y.-H. Kim, Y. Qi, T.-W. Lee, Universal energy level tailoring of self-organized hole extraction layers in organic solar cells and organic-inorganic hybrid perovskite solar cells. *Energ. Environ. Sci.* **9**, 932–939 (2016).

48. H. Kim, K.-G. Lim, T.-W. Lee, Planar heterojunction organometal halide perovskite solar cells: Roles of interfacial layers. *Energ. Environ. Sci.* **9**, 12–30 (2016).

49. Z. Huang, Y. Hao, Y. Li, H. Hu, C. Wang, A. Nomoto, T. Pan, Y. Gu, Y. Chen, T. Zhang, W. Li, Y. Lei, N. H. Kim, C. Wang, L. Zhang, J. W. Ward, A. Maralani, X. Li, M. F. Durstock, A. Pisano, Y. Lin, S. Xu, Three-dimensional integrated stretchable electronics. *Nat. Electron.* **1**, 473–480 (2018).

50. J. W. Lee, R. Xu, S. Lee, K. I. Jang, Y. Yang, A. Banks, K. J. Yu, J. Kim, S. Xu, S. Ma, S. W. Jang, P. Won, Y. Li, B. H. Kim, J. Y. Choe, S. Huh, Y. H. Kwon, Y. Huang, U. Paik, J. A. Rogers, Soft, thin skin-mounted power management systems and their use in wireless thermography. *Proc. Natl. Acad. Sci. U.S.A.* **113**, 6131–6136 (2016).

51. S. Xu, Y. Zhang, Jia, L, K. E. Mathewson, K. I. Jang, J. Kim, H. Fu, X. Huang, P. Chava, R. Wang, S. Bhole, L. Wang, Y. J. Na, Y. Guan, M. Flavin, Z. Han, Y. Huang, J. A. Rogers, Soft microfluidic assemblies of sensors, circuits, and radios for the Skin. *Science* **344**, 70–74 (2014).

52. D.-H. Kim, J. Viventi, J. J. Amsden, J. Xiao, L. Vigeland, Y.-S. Kim, J. A. Blanco, B. Panilaitis, E. S. Frechette, D. Contreras, D. L. Kaplan, F. G. Omenetto, Y. Huang, K.-C. Hwang, M. R. Zakin, B. Litt, J. A. Rogers, Dissolvable films of silk fibroin for ultrathin conformal bio-integrated electronics. *Nat. Mater.* **9**. 511–517 (2010).

53. Y. F. Yin, M. Li, Li Y. H., J. Z. Song, Skin pain sensation of epidermal electronic device/skin system considering non-Fourier heat conduction. *J. Mech. Phys. Solids* **138**, 103927 (2020).

54. J. P. Zhang, Y. H. Li, Y. F. Xing, Theoretical and experimental investigations of transient thermo-mechanical analysis on flexible electronic devices. *Int. J. Mech. Sci.* **160**, 192–199 (2019).

55. Y. Liu, L. Zhao, R. Avila, C. Yiu, T. Wong, Y. Chan, K. Yao, D. Li, Y. Zhang, W. Li, Z. Xie, X. Yu, Epidermal electronics for respiration monitoring via thermo-sensitive measuring. *Mater. Today Phys.* **13**, 100199 (2020).

56. S. Xu, Y. Zhang, J. Cho, J. Lee, X. Huang, L. Jia, J. A. Fan, Y. Su, J. Su, H. Zhang, H. Cheng, B. Lu, C. Yu, C. Chuang, T.-i. Kim, T. Song, K. Shigeta, S. Kang, C. Dagdeviren, I. Petrov, P. V. Braun, Y. Huang, U. Paik, J. A. Rogers, Stretchable batteries with self-similar serpentine interconnects and integrated wireless recharging systems. *Nat. Commun.* **4**. 1543 (2013).

57. Y. Kim, A. Chortos, W. Xu, Y. Liu, J. Y. Oh, D. Son, J. Kang, A. M. Foudeh, C. Zhu, Y. Lee, S. Niu, J. Liu, R. Pfattner, Z. Bao, T. W. Lee, A bioinspired flexible organic artificial afferent nerve. *Science* **360**, 998–1003 (2018).

58. T. Wang, H. Yang, D. Qi, Z. Liu, P. Cai, H. Zhang, X. Chen, Mechano-based transductive sensing for wearable healthcare. *Small* **14**, 1702933 (2018).

59. Y. J. Ma, Y. C. Zhang, S. S. Cai, Z. Y. Han, X. Liu, F. L, Wang, Y. Cao, Z. H. Wang, H. F. Li, Y. H. Chen, X. Feng, Flexible hybrid electronics for digital healthcare. *Adv. Mater.* **32**, 1902062 (2020)

60. Y. J. Ma, J. Choi, A. Hourlier-Fargette, Y. G. Xue, H. U. Chung, J. Y. Lee, X. F. Wang, Z. Q. Xie, D. Kang, H. L. Wang, S. Han, S. K. Kang, Y. Kang, X. Yu, M. J. Slepian, M. S. Raj, J. B. Model, X. Feng, R. Ghaffari, J. A. Rogers, Y. Huang, Relation between blood pressure and pulse wave velocity for human arteries. *Proc. Natl. Acad. Sci. U.S.A.* **115**, 11144–11149 (2018).

61. H. Li, Y. Ma, Z. Liang, Z. Wang, Y. Cao, Y. Xu, H. Zhou, B. Lu, Y. Chen, Z. Han, S. Cai, X. Feng, Wearable skin-like optoelectronic systems with suppression of motion artifact for cuff-less continuous blood pressure monitor. *Natl. Sci. Rev.* **7**, 849–862 (2020).

62. Y. Chen, S. Lu, S. Zhang, Y. Li, Z. Qu, Y. Chen, B. Lu, X. Wang, X. Feng, Skin-like biosensor system via electrochemical channels for noninvasive blood glucose monitoring. *Sci. Adv.* **3**, e1701629 (2017).

63. H. Li, Y. Xu, X. Li, Y. Chen, Y. Jiang, C. Zhang, B. Lu, J. Wang, Y. Ma, Y. Chen, Y. Huang, M. Ding, H. Su, G. Song, Y. Luo, X. Feng, Epidermal inorganic optoelectronics for blood oxygen measurement. *Adv. Healthc. Mater.* **6**, 1601013 (2017).

64. C. Wang, X. Li, H. Hu, L. Zhang, Z. Huang, M. Lin, Z. Zhang, Z. Yin, B. Huang, H. Gong, S. Bhaskaran, Y. Gu, M. Makihata, Y. Guo, Y. Lei, Y. Chen, C. Wang, Y. Li, T. Zhang, Z. Chen, A. P. Pisano, L. Zhang, Q. Zhou, S. Xu, Monitoring of the central blood pressure waveform via a conformal ultrasonic device. *Nat. Biomed. Eng.* **2**, 687–695 (2018).

65. M. J. Weber, Y. Yoshihara, A. Sawaby, J. Charthad, T. C. Chang, A. Arbabian, A miniaturized single-transducer implantable pressure sensor with time-multiplexed ultrasonic data and power links. *IEEE J. Solid-State Circuits* **53**, 1089–1101 (2018).

66. M. M. Ghanbari, D. K. Piech, K. Shen, S. F. Alamouti, C. Yalcin, B. C. Johnson, J. M. Carmena, M. M. Maharbiz, R. Muller, A sub-mm 3 ultrasonic free-floating implant for multi-mote neural recording. *IEEE J. Solid-State Circuits* **54**, 3017–3030 (2019).

67. C. Shi, T. Costa, J. Elloian, Y. Zhang, K. L. Shepard, A 0.065-mm^3 monolithically-integrated ultrasonic wireless sensing mote for real-time physiological temperature monitoring. *IEEE Trans. Biomed. Circuits Syst.* **14**, 412–424 (2020).

68. R. Hinchet, H. J. Yoon, H. Ryu, M. K. Kim, E. K. Choi, D. S. Kim, S. W. Kim, Transcutaneous ultrasound energy harvesting using capacitive triboelectric technology. *Science* **365**, 491–494 (2019).

69. S. Sonmezoglu, J. R. Fineman, E. Maltepe, M. M. Maharbiz, Monitoring deep-tissue oxygenation with a millimeter-scale ultrasonic implant. *Nat. Biotechnol.* **39**, 855–864 (2021).

70. V. Arumugam, M. Naresh, R. Sanjeevi, Effect of strain rate on the fracture behaviour of skin. *J. Biosci.* **19**, 307–313 (1994).

71. Z. Jia, C. Yang, J. Jiao, X. Li, D. Zhu, Y. Yang, J. Yang, Y. Che, Y. Lu, X. Feng, Rhein and polydimethylsiloxane functionalized carbon/carbon composites as prosthetic implants for bone repair applications. *Biomed. Mater.* **12**, 045004 (2017).

72. M. C. Bélanger, Y. Marois, Hemocompatibility, biocompatibility, inflammatory and in vivo studies of primary reference materials low-density polyethylene and polydimethylsiloxane: A review. *J. Biomed. Mater. Res.* **58**, 467–477 (2001).

73. W. Qian, X. Hu, W. He, R. Zhan, M. Liu, D. Zhou, Y. Huang, X. Hu, Z. Wang, G. Fei, J. Wu, M. Xing, H. Xia, G. Luo, Polydimethylsiloxane incorporated with reduced graphene oxide (rGO) sheets for wound dressing application: Preparation and characterization. *Colloids Surf. B Biointerfaces* **166**, 61–71 (2018).

74. H. J. Brandon, K. L. Jerina, C. J. Wolf, V. L. Young, Biodurability of retrieved silicone gel breast implants. *Plast. Reconstr. Surg.* **111**, 2295–2306 (2003).

75. P. Gutruf, R. T. Yin, K. B. Lee, J. Ausra, J. A. Brennan, Y. Qiao, Z. Xie, R. Peralta, O. Talarico, A. Murillo, S. W. Chen, J. P. Leshock, C. R. Haney, E. A. Waters, C. Zhang, H. Luan, Y. Huang, G. Trachiotis, I. R. Efimov, J. A. Rogers, Wireless, battery-free, fully implantable multimodal and multisite pacemakers for applications in small animal models. *Nat. Commun.* **10**, 5742 (2019).

76. V. T. Rathod, A review of acoustic impedance matching techniques for piezoelectric sensors and transducers. *Sensors* **20**, 4051 (2020).

77. B. H. Waters, A. P. Sample, P. Bonde, J. R. Smith, Powering a ventricular assist device (VAD) with the free-range resonant electrical energy delivery (FREE-D) system. *Proc. IEEE* **100**, 138–149 (2012).

78. A. Abid, J. M. O'Brien, T. Bensel, C. Cleveland, L. Booth, B. R. Smith, R. Langer, G. Traverso, Wireless power transfer to millimeter-sized gastrointestinal electronics validated in a swine model. *Sci. Rep.* **7**, 46745 (2017).

79. Y. Shih, T. Shen, B. Otis, A 2.3 μ W wireless intraocular pressure/temperature monitor. *IEEE J. Solid-State Circuits* **46**, 2592–2601 (2011).

80. H. U. Chung, B. H. Kim, J. Y. Lee, J. Lee, Z. Xie, E. M. Ibler, K. H. Lee, A. Banks, J. Y. Jeong, J. Kim, C. Ogle, D. Grande, Y. Yu, H. Jang, P. Assem, D. Ryu, J. W. Kwak, M. Namkoong, J. B. Park, Y. Lee, D. H. Kim, A. Ryu, J. Jeong, K. You, B. Ji, Z. Liu, Q. Huo, X. Feng, Y. Deng, Y. Xu, K.-I. Jang, J. Kim, Y. Zhang, R. Ghaffari, C. M. Rand, M. Schau, A. Hamvas, D. E. Weese-Mayer, Y. Huang, S. M. Lee, C. H. Lee, N. R. Shanbhag, A. S. Paller, S. Xu, J. A. Rogers, Binodal, wireless epidermal electronic systems with in-sensor analytics for neonatal intensive care. *Science* **363**, eaau0780 (2019).

81. X. Liu, J. L. Berger, A. Ogirala, M. H. Mickle, A touchprobe method of operating an implantable RFID tag for orthopedic implant identification. *IEEE Trans. Biomed. Circuits Syst.* **7**, 236–242 (2013).

82. M Kiani, K. Y. Kwon, F. Zhang, K. Oweiss, M. Ghovanloo, Evaluation of a closed loop inductive power transmission system on an awake behaving animal subject. *Annual Int. Conf. IEEE Eng. Med. Biol. Soc.* **2011**, 7658–7661 (2011).

83. J. Charthad, M. J.Weber, T. C. Chang, A. Arbabian, A mm-sized implantable medical device (IMD) with ultrasonic power transfer and a hybrid bi-directional data link. *IEEE J. Solid-State Circuits* **50**, 1741–1753 (2015).

84. S. Ozeri, D. Shmilovitz, S. Singer, C. C. Wang, Ultrasonic transcutaneous energy transfer using a continuous wave 650 kHz Gaussian shaded transmitter. *Ultrasonics* **50**, 666–674 (2010).
